# Mutation Spectrum of Cancer-Associated Genes in Patients With Early Onset of Colorectal Cancer

**DOI:** 10.3389/fonc.2019.00673

**Published:** 2019-08-02

**Authors:** Gulnur Zhunussova, Georgiy Afonin, Saltanat Abdikerim, Abai Jumanov, Anastassiya Perfilyeva, Dilyara Kaidarova, Leyla Djansugurova

**Affiliations:** ^1^Laboratory of Molecular Genetics, Institute of General Genetics and Cytology, Almaty, Kazakhstan; ^2^Center of Thoracic and Abdominal Oncology, Kazakh Institute of Oncology and Radiology, Almaty, Kazakhstan; ^3^Department of Molecular Biology and Genetics, Al-Farabi Kazakh National University, Almaty, Kazakhstan; ^4^Department of Oncology, Asfendiyarov Kazakh National Medical University, Almaty, Kazakhstan

**Keywords:** early-onset CRC, next-generation sequencing, pathogenic mutation, primary multiple tumors, family history of cancer

## Abstract

**Background:** Colorectal cancer (CRC) incidence is rising worldwide, as well as in the Republic of Kazakhstan, while its occurrence is also increasing in the younger population. Hereditary forms associated with the development of colon and rectal cancer and early-onset CRC have never been studied in the population of Kazakhstan. The aim of this research was to investigate the spectrum of CRC-related gene mutations to determine which mutations cause early onset of CRC in the Kazakhstan population.

**Methods:** The study included 125 unrelated patients from Kazakhstan (range 17–50 years in age) with early onset CRC. Genomic DNA was obtained from peripheral blood of the patients. Next-generation sequencing was performed using the TruSightCancer Kit on the MiSeq platform. The Studio Variant was used to annotate and interpret genetic variants.

**Results:** Bioinformatics analysis of Next-generation sequencing data revealed 11,152 variants from 85 genes, of them, 3,790 missense, 6,254 synonymous variants, 44 3′UTR variants, 10 frameshift variants, five stop-gain variants, four in-frame deletions, two splice donors, one splice acceptor variant, and 1,042 intron or non-coding variants. *APC, BRCA2/1, ALK, BRIP1, EGFR, FANCA, FANCD2, FANCI, HNF1A, MEN1, NSD1, PMS2, RECQL4, RET, SLX4, WRN*, and *XPC* genes mutated most often. According to the ACMG guidelines and LOVD/ClinVar databases, 24 variants were pathogenic (10 frameshifts, five missenses, five stop-gain, one in-frame deletion, and three splice-site mutations), and 289 were VUS with population frequency <1%, 131 of them were attributed as deleterious. In the study, 50% of all pathogenic mutations found in Kazakhstani patients with early CRC onset were identified in the subgroups with a family history of CRC and primary multiple tumors. In *APC*, pathogenic mutations were most often (21%).

**Conclusion:** Pathogenic and likely pathogenic mutations were found in 20 (16%) out of 125 patients. Eight novel pathogenic mutations detected in *FANCI, APC, BMPR1, ATM*, and *DICER1* genes have not been reported in previous literature. Given the high frequency and wide spectrum of mutations, NGS analysis must be carried out in families with a history of CRC/CRC-related cancers with the purpose to identify cause-effective mutations, clarify the clinical diagnosis, and prevent the development of the disease in other family members.

## Introduction

Currently, colorectal cancer (CRC) is one of the most common types of cancer, with more than 1.8 million new cases and 800,000 deaths registered each year in the world. According to GLOBOCAN, 1,849,518 cases of CRC (10.2% of all cancers) were registered in the world in 2018 (1). According to IARC global data, CRC ranks 3rd in the structure of cancer incidence in men, and 2nd in women (2).

Considering current trends and the dynamics of the demographic situation (population growth and population aging), by 2030, WHO predicts an increase of 77% in global CRC incidence (to 2.2 million new cases per year) and 80% mortality (to 1.1 million deaths from CRC per year). Worldwide, CRC incidence is more influenced by a family history of cancer (FHC) and social (mainly environmental) factors, than by geographical factors (3).

CRC frequency positively and strongly correlates with age. CRC likelihood in the general population increases after age 40, with a dramatic increase after age 50; 90% of CRC cases occur in people over the age of 50 (4).

Colorectal cancer (CRC) has a complex nature which is a sequential interaction of environmental and genetic factors. Sporadic CRC occurs pre-dominantly in the elderly and at a senile age and accounts for nearly 80% of all cases. Early-onset CRC is mostly associated with hereditary syndromes as they pre-dispose the development of CRC as a result of mutations in well-studied genes. Currently, several specific conditions are considered as etiological factors: hereditary non-polyposis colorectal cancer (HNPCC, also known as Lynch syndrome) (5), familial adenomatous polyposis (FAP) syndrome, other rarer syndromes and syndromic associations (Peutz-Jeghers, Gardner-Turner, Bannayan–Riley–Ruvalcaba, Bloom's disease, Tourette's syndrome), inflammatory bowel disease, infection caused by human papillomavirus (squamous cell carcinoma of rectum and anal), and HIV. Lynch syndrome and FAP are the most common known causes of CRC development (6–12).

In the Republic of Kazakhstan, 325,606 patients were registered with malignant tumors from 2008 to 2018. Twenty-eight thousand (8.6%) of them were diagnosed with CRC, including 14,833 (52.9%) cases of colon cancer, and 13,185 (47.1%) cases of rectal cancer. The number of detected CRC cases grew by an average of 845 cases per year, and the CRC incidence rating among other cancers has increased from 5th to 4th place. In the last 10 years, 3,121 (11.1%) of all new CRC patients were younger than 50. In line with a growing CRC incidence among younger adults in Kazakhstan, the number of early-onset CRC patients before age 50 has increased by 14.8%, from 305 cases in 2008 to 350 cases in 2017.

Unfortunately, there are no statistics on hereditary types of CRC in Kazakhstan. For this reason, we are collecting biomaterials and family histories of patients with early-onset CRC.

According to the classic CRC carcinogenesis model, several key genetic changes are required for tumor growth initiation and progression. The earliest genetic trigger is the *APC* gene inactivation. The mutations in other suppressor genes (*SMAD2, SMAD4, DCC*, and *TP53*), oncogenes (*KRAS, BRAF*), and some other genes contribute to neoplastic transformation in foci of the altered colon mucosa which in turn leads to further degeneration toward malignancy. These mutations may be accompanied by the deregulation of the expression of oncogenes and/or tumor suppressor genes and subsequent epigenetic modifications of their promoter regions. However, other genetic changes are also involved in the development of CRC. Thus, each hereditary syndrome is associated with a specific spectrum of gene mutations (13–15).

The results of genetic analysis of cancer patients indicate an annual increase in the number of genetic changes of prognostic, diagnostic, or clinical significance. Advances in genome and epigenome analysis provide new information on hereditary CRC variants. This creates a need for robust clinical tests able to simultaneously detect multiple genetic changes. Next-generation sequencing (NGS) fully meets this growing demand thanks to the high generating ability of DNA analysis and good clinical throughput. NGS platforms allow for the identification of cause-effect mutations in the early onset of CRC. These mutations have already been described and included in widely used databases such as ClinVar (16), LOVD/InSIGHT (available via the Leiden Open-Source Variation Database (LOVD) http://www.lovd.nl/3.0/home or https://www.insight-group.org/variants/database/), the Catalog of Somatic Mutations in Cancer (COSMIC, http://cancer.sanger.ac.uk/cancergenome/projects/cosmic/) together with the novel, previously undescribed mutations of key CRC genes.

Therefore, we used multiple gene panels and NGS to screen patients from Kazakhstan with early-onset CRC for potential pathogenic mutations.

## Materials and Methods

### Study Cohort

The study was conducted on 125 patients aged between 17 and 50 from the Republic of Kazakhstan with early-onset of CRC. All the patients signed the informed consent and filled in the detailed questionnaire which included questions related to the FHC. The consent form and the questionnaire were approved by the IRB/IEC of Kazakh Institute of Oncology and Radiology (KazIOR) and Asfendiyarov Kazakh National Medical University (Almaty, Kazakhstan). In cases observed before 2017, cancer staging was done according to the 7th edition of the AJCC Cancer Staging System; in cases observed after 2017—according to the 8th edition of the AJCC Cancer Staging System (The AJCC Cancer Staging Manual, Eighth Edition, https://cancerstaging.org/references-tools/deskreferences/Pages/8EUpdates.aspx#).

### DNA Preparation

Genomic DNA (gDNA) was isolated from EDTA treated peripheral blood samples using the GeneJET Genomic DNA Purification Kit (*Thermo Scientific, USA*). Qualitative and quantitative characteristics of the DNA samples were evaluated by the spectrophotometer (*Eppendorf Biophotometer plus, Germany*) and fluorometer (*Quantus*™ *Promega, USA*). At least 50 ng of double-stranded nuclear DNA was obtained per sample.

### NGS—Library Preparation and Sequencing

Next-generation sequencing (NGS) was performed using TruSight Rapid Capture Kit (Illumina, USA) in combination with the TruSight Cancer Sequencing Panel (Illumina, USA) according to the manufacturer's recommendations.

DNA libraries with 4 mM molarities were subjected to clustering using a standard flow cell and were sequenced on the MiSeq platform (Illumina) using the MiSeq Reagent Kit v3 (600 cycles).

### NGS—Bioinformatics Data Analysis

Bioinformatics data analysis was carried out using two methodological approaches with different algorithms of analysis. In the first approach, the NGS data were analyzed using MiSeq Reporter v.3.0 software (Illumina). This software allowed mapping and aligning the sequenced sequences and comparing them with the reference sequence of the human genome (GRCH37/hg19) using the Burrows-Wheeler Aligner (BWA) algorithm. Search and detection of variants for specific regions of the genome were carried out using the Genome Analysis Toolkit (GATK, Broad Institute, Cambridge, USA).

In the second approach, various bioinformatics methods and software packages were used to optimize the workflow. The mapping and alignment of a sequence read against the human reference genome of the GRCH37/hg19 version were conducted using the Bowtie2 algorithm with a very sensitive local parameter. FastQC and MultiQC were used to evaluate the quality of reads of sequences. Picard Tools and SAM Tools/BCF tools were used to convert SAM files into BAM files, sort, and index the mapped reads, remove intermediate files, merge the BAM files, and identify duplicates. Java Runtime Environment and R Bioconductor were used to execute software scripts. GATK was used to re-align the mapped reads around the areas with insertions/deletions. GATK “Haplotype caller” strategy was used to filter and detect genomic variants. Finally, all VCF (variant call format) files containing only alternative variants obtained by GATK were imported into the Variant Studio 3.0 software (Illumina). In the study, only the variants identified by both approaches were considered.

### Annotation, Interpretation, and Classification of Variants

Variant Studio was used to annotate and interpret genetic variants. A Q-score of 30, corresponding to an error rate of 0.1%, was selected as the acceptance threshold value. The following filtration parameters were set to exclude poor quality nucleotide variants: read depth >50×, alternative read depth >20×, quality value >100. Variants that matched the filtering parameters were selected for the study.

Genetic variants were annotated in accordance with the nomenclature of the Human Genome Variation Society (HGVS) (17); the interpretation was done using the Single Nucleotide Polymorphism Database (dbSNP, http://www.ncbi.nlm.nih.gov/projects/SNP/), ClinVar database, InSIGHT/LOVD database, and COSMIC. Integrative Genomics Viewer was applied to visualize the variants (18, 19).

Identified variants were categorized in accordance with the guidelines of the American College of Medical Genetics and Genomics (ACMG) (20) as pathogenic, likely pathogenic, uncertain significance (VUS), benign, and likely benign. Pathogenic mutations included those resulting in a premature stop codon (frameshift mutations and nonsense), variants with uncorrected splicing and variants affecting protein function according to the results of functional studies. Novel mutations such as frameshift mutations and nonsense mutations leading to protein truncation, as well as mutations without well-established functional studies, were classified as likely pathogenic. Synonymous and intronic variants not affecting splicing were regarded to be benign/probably benign, as well as the variants with a minor allele frequency (MAF) found in 5% or more of the general population according to the 1000 Genomes database (1000G) (21), the Exome Sequencing Project database (ESP6500, https://esp.gs.washington.edu) or the Exome Aggregation Consortium database (ExAC) (20). The remaining variants with no functional data and not fulfilling the criteria of classification as pathogenic/likely pathogenic or benign/likely benign, or with conflicting evidence of their benign and pathogenic nature, were defined as VUS.

*In silico*, bioinformatics tools such as Sorting Intolerant From Tolerant (SIFT) (22) and Polymorphism Phenotyping-2 (PolyPhen-2, http://genetics.bwh.harvard.edu/pph2/) (23) were used to predict potential pathogenic effects of missense variants on protein structure and function. The variants that matched the following criteria: a SIFT score of <0.05 and a PolyPhen-2 score between 0.95 and 1.0, were considered to be strongly suspected of being deleterious (24).

### Statistical Analysis

Demographics, clinical-pathological characteristics, and personal and family history variables were analyzed. Descriptive statistics were summarized as frequency distributions for categorical variables and as means for continuous variables. Wilcoxon rank sum tests were used to compare continuous variables, and the chi-square test was performed to compare categorical variables. The results were considered statistically significant at a *p* < 0.05. All statistical tests were two-sided at the 5% level of significance. Data were processed and analyzed by using R software, version 3.6.0 (W.N. Venables, D.M. Smith, and the R Core Team).

### Data Availability

Sequencing data have been deposited at the NCBI SRA archive with BioProject record PRJNA548114 and SRA accession SUB5747000.

## Results

### Studied Cohort

Study cohort included 62 (49.6%) men and 63 (50.4%) women with early onset CRC. Th ethnic composition of the patient group included Kazakhs−61 (48.8%), Russians−36 (28.8%), Uyghurs−7 (5.6%), Azerbajanians−3 (2.4%), Koreans−3 (2.4%), Germans−3 (2.4 %), Ukrainians−2 (1.6%), Tatars−2 (1.6%), Belarusians, Dungans, Turks−1 patient each (0.8% each), patients with mixed ethnic background−5 (4%).

The median age of patients at CRC diagnosis was 40.7 (39.6 in men, 41.7 in women). 25 patients (20.1% of the early CRC onset cohort, of them, 15 men and 10 women) were diagnosed with CRC before the age of 35 (median age 29.08 ± 4.75 for both sexes; 28.4 ± 45.2 in men and 30.1 ± 5.15 in women).

In terms of clinical pathology, patients with early CRC onset differed by TNM staging, tumor localization, and histological types. The pre-dominant histological type was moderately differentiated adenocarcinoma detected in 81 (64.8%) cases. Half of the patients (57.2%) were diagnosed with stage III cancer. The rectum was a more common tumor site in both men and women compared to other sites. No significant statistical difference was found in all clinical characteristics between males and females (Table 1).

**Table 1:** Clinicopathological features of patients with early onset of CRC.

**Primary tumor location**	**Total (%)**	**Male (%)**	**Female (%)**	***P* (male vs. female)**
Right-sided colon	12 (9.6%)	6 (9.8%)	6 (9.5%)	1.00
Left-sided colon	37 (29.6%)	15 (24.2%)	22 (35.0%)	0.25
Rectosigmoid	8 (6.4%)	4 (6.5%)	4 (6.3%)	1.00
Rectum	58 (46.4%)	34 (54.8%)	24 (38.1%)	0.19
Primary multiple tumors (PMTs) localized in various cancer site including various CRC site	10 (8.0%)	3 (4.8%)	7 (11.1%)	0.20
**Histology**	**Total (%)**	**Male (%)**	**Female (%)**	
Well-differentiated adenocarcinoma	9 (7.2%)	4 (3.2%)	5 (4%)	0.74
Moderately differentiated adenocarcinoma	81 (64.8%)	42 (33.6%)	39 (31.2%)	0.74
Poor-differentiated adenocarcinoma	8 (6.4%)	3 (2.4%)	5 (4%)	0.48
Undifferentiated adenocarcinoma	1 (0.8%)	–	1 (0.8%)	–
Mucous adenocarcinoma	15 (12.0%)	5 (4%)	10 (8%)	0.19
The degree of differentiation was not indicated	8 (6.4%)	5 (4%)	3 (2.4%)	0.48
Others (Squamous-cell carcinoma, signet-ring and dark-cell adenocarcinomas)	3 (2.4%)	3 (2.4%)	–	–
**TNM stage**	**Total (%)**	**Male (%)**	**Female (%)**	
I	5 (4.0%)	2 (3.2%)	3 (4.8%)	0.65
II	34 (27.2%)	15 (24.2%)	19 (30.1%)	0.49
III	57 (45.6%)	26 (42.0%)	31 (49.2%)	0.51
IV	29 (23.2%)	19 (30.6%)	10 (15.9%)	0.09
**Family history of cancer**				
With FHC	19 (15.2%)	9 (47.4%)	10 (52.6)	0.82
Without FHC	106 (84.8%)	53 (50%)	53 (50%)	1.00
**Syndromic CRC**	6 (4.8%)	4 (3.2%)	3 (2.4%)	0.70

*There were no statistically significant differences in all clinical characteristics between males and females (p > 0 05 for all characteristics)*.

**Figure 1:**
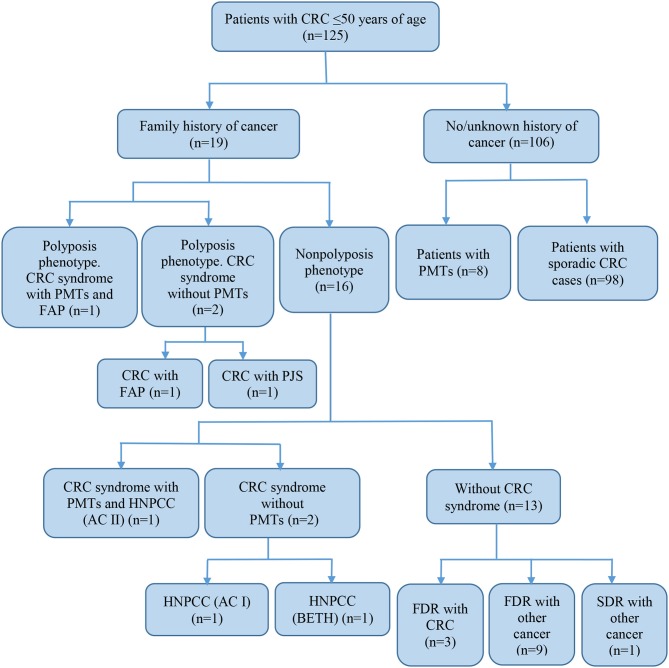
Characteristics of subjects with early-onset CRC. CRC, colorectal cancer; FAP, familial adenomatous polyposis; PJS, Peutz-Jeghers syndrome; HNPCC, hereditary non-polyposis colorectal cancer; AC, Amsterdam I or II criteria; BETH, Bethesda criteria; PMTs, primary multiple tumors; FDR, first-degree relative; SDR, second-degree relative.

Of all the subjects, 19 (15.2%) patients with a family history of cancer (FHC), 6 had the CRC syndrome (Table 2). The characterization of all the patients with early onset CRC is shown in Figure 1.

**Table 2:** Characteristics of the patients with a family history of cancer.

**No**	**Patient ID**	**Gender**	**Age**	**Ethnicity**	**Proband's diagnosis**	**Family history of cancer**
1	CRC2	Male	26	Turk	FAP; Sigmoid colon cancer	Family members with FAP and CRC
2	CRC613	Female	39	Father: Russian Mother: Kazakh	FAP; Primary multiple synchronous cancer. Cancer splenic flexure; Rectal cancer	Family member with FAP and CRC
3	CRC607	Male	18	Kazakh	HNPCC (Amsterdam II criteria)^*^ Ascending colon cancer	Mat.great-grandmother: endometrial cancer; Mat. grandmother: gastric cancer; Mat. Aunt (sister of mother): CRC; One aunt of mother: gastric cancer; Other aunt of mother: CRC.
4	CRC608	Male	33	Russian	HNPCC (Amsterdam criteria I)^*^ Rectal cancer	Father, father's younger brother, and sister: CRC. Paternal grandmother: unknown cancer
5	CRC629	Female	47	Russian	HNPCC (Bethesda criteria)^*^ Primary multiple metachronous cancer. Rectosigmoid colon cancer; BC; OC	Mat. grandmother and mother: OC; Mat.great-grandmother:
6	CRC596	Male	28	German	Peutz–Jeghers syndrome. Rectal cancer	Family members with CRC and diffuse polyposis
7	CRC368	Female	39	Kazakh	Rectal cancer	Mother: CRC
8	CRC584	Male	25	Kazakh	Rectal cancer	Father: gastric cancer
9	CRC587	Male	46	Kazakh	Rectal cancer	Father: kidney cancer
10	CRC621	Female	47	Tatar	Rectal cancer	Mother: breast cancer, Father: cancer of the large duodenal papilla
11	CRC622	Male	43	Russian	Caecum cancer	Mother: cancer of the ascending colon, Maternal grandmother–gastric cancer
12	CRC625	Female	48	Belarus	Sigmoid colon cancer	Father: laryngeal cancer
13	CRC626	Female	36	Kazakh	Rectal cancer	Mother: endometrial cancer
14	CRC627	Male	46	Russian	Rectal cancer	Paternal grandfather: lung cancer; Paternal grandmother: breast cancer
15	CRC629	Female	47	Russian	Primary multiple metachronous cancer: OC, BC, rectosigmoid colon cancer	Maternal grandmother: ovarian cancer; Mother: ovarian cancer
16	CRC632	Male	32	Kazakh	Rectal cancer	Father: gastric cancer
17	CRC636	Female	46	Kazakh	Rectal cancer	Father: prostate cancer
18	CRC639	Female	49	Russian	Sigmoid colon cancer	Mother: rectal cancer
19	CRC335	Female	47	Russian	Rectosigmoid colon cancer	Father: prostate cancer

* *Characterized according to Silva et al. (5)*.

### NGS Sequencing Results

A total of 125 unrelated patients with early onset CRC were analyzed by NGS using the TruSight Cancer Sequencing Panel which targeted a set of 94 genes known to play a role in cancer pre-disposition. Bioinformatics analysis of NGS data has revealed 11,152 variants (Supplementary Table 1) in 85 genes, of them, 3,790 missense, 6,254 synonymous variants, 44 3′UTR variants, 10 frameshift variants, five stop-gain variants, four in-frame deletions, two splice donors, one splice acceptor variant, and 1,042 intron or non-coding variants. *APC, BRCA2/1, ALK, BRIP1, EGFR, FANCA, FANCD2, FANCI, HNF1A, MEN1, NSD1, PMS2, RECQL4, RET, SLX4, WRN*, and *XPC* genes mutated most often.

The approach to the interpretation of NGS data described in the corresponding databases and guidelines mentioned in the Materials and Methods section has revealed 24 pathogenic/likely pathogenic mutations. 472 variants were classified as VUS; 10,656 variants—as benign/likely benign.

### Pathogenic and Likely Pathogenic Variants

Twenty-four pathogenic and probable pathogenic mutations were detected in 20 patients (16% of the entire group of patients) with cancer of the cecum, ascending colon, hepatic flexure of the colon, splenic flexure of the colon, sigmoid colon, rectosigmoid colon, and rectum. Of them, five patients had a family history of FAP, CRC, gastric cancer, or ovarian cancer. Six patients (four cases of synchronous cancers and two cases of metachronous cancer) had primary multiple tumors (PMTs). Out of those pathogenic and likely pathogenic variants, five were detected in the *APC* gene, three in *FANCI*, three in *BRCA2*, two in *BRCA1*, two in *MLH1*, and one mutation each, in *MSH6, MUTYH, BLM, NBN, ATM, BMPR1A, CHEK2, AIP*, and *DICER1* genes. As shown in Table 3, the analysis of mutation type has revealed 10 frameshift mutations, five missense mutations, five stop-gain mutations, one in-frame deletion, and three mutations involving uncorrected splicing. All detected pathogenic and likely pathogenic mutations were in the heterozygous state. In total, 23 mutations were unique, eight of them represented novel variants. Those new mutations have not been previously mentioned in the LOVD and ClinVar databases and have not been described in publications. All mutations were rare, and for 17 of them, frequency data was not available in the 1000G, ESP6500 and ExAC databases (Table 3). 62.5% of the mutations were available in the dbSNP database, 29.1% in the COSMIC catalog, and 38.4% in the ClinVar and/or LOVD databases. Multiple alterations of genes were noticed in three patients. Three frameshift variants including two novel variants (c.907delA in *APC* gene and c.6743dupA in *ATM*) and one previously reported mutation (c.657_661delACAAA in *NBN*) were detected in one patient (male, Kazakh) at the age of 40 with rectum cancer. Two more patients (one male at the age of 26 with FAP and sigmoid colon cancer and one male at the age of 46 with ascending colon cancer and rectum cancer) had one novel frameshift variant each (*FANCI* c.3340delA and *BMPR1A* c.152delC, respectively) in combination with other previously reported frameshift variants (APC c.3613delA and *CHEK2* c.599T > C, respectively, Table 3).

**Table 3:** Pathogenic and likely pathogenic variants identified by TruSight cancer sequencing panel in 20 patients with early-onset CRC.

**Patient ID**	**Clinical features (age at diagnosis/gender)**	**Ethnicity**	**Family history**	**Gene**	**Geno type**	**Mutation type**	**dbSNP ID**	**HGVSc**	**HGVSp**	**Database**	**Population frequency**		
											**1000G**	**Esp6500**	**ExAC**
CRC2	FAP; Sigmoid colon cancer (26/M)	Turk	A family member with FAP and CRC	*APC*	Het	Frameshift variant	NA	c.3613delA	p.Ser1205 AlafsTer60	LOVD	NA	NA	NA
				*FANCI*	Het	Frameshift variant	NA	c.3340delA	p.Thr1114 ProfsTer50	Novel	NA	NA	NA
CRC629	Primary multiple metachronous cancer. Rectosigmoid colon cancer; BC; OC (47/F)	Russian	Mat. grandmother and mother: OC	*BRCA1*	Het	Frameshift variant	rs39750724; rs80357906	c.5329dupC (known as c.5382insC)	p.Gln1777 ProfsTer74	LOVD, ClinVar	NA	NA	0.02
CRC622	Cecum cancer (43/M)	Russian	Mother: ascending colon cancer; Mat.grandmother: gastric cancer	*MLH1*	Het	Missense	rs267607706	c.114C > G	p.Asn38Lys	LOVD, ClinVar	NA	NA	NA
CRC596	PJS (20/M); Rectum Cancer (25)	German	Family members with CRC and diffuse polyposis	*APC*	Het	Stop-gain	rs1060503299	c.3827C > G	p.Ser1276Ter	LOVD, ClinVar^#^	NA	NA	NA
CRC613	FAP; Primary multiple synchronous cancer. Cancer splenic flexure; Rectum Cancer (39/F)	Father: Russian; Mother: Kazakh	A family member with FAP and CRC	*APC*	Het	Stop-gain	NA	c.4128T > G	p.Tyr1376Ter	Novel^#^	NA	NA	NA
CRC624	Primary multiple synchronous cancer. Sigmoid colon cancer; Hepatic flexure of the colon cancer (46/M)	Russian	No family history	*MLH1*	Het	In-frame deletion	rs121912962; rs587782285	c.1852_1854 delAAG	p.Lys618del	LOVD, ClinVar^#^	NA	NA	NA
CRC344	Primary multiple synchronous cancer. Ascending colon cancer; Rectum cancer (46/F)	German	No family history	*BMPR1A*	Het	Frameshift variant	NA	c.152delC	p.Ala51 AspfsTer9	Novel	NA	NA	NA
				*CHEK2*	Het	Missense	rs17879961	c.599T>C	p.Ile200Thr	ClinVar^#^	0.1	0.16	0.41
CRC553	Primary multiple metachronous cancer. Sigmoid colon cancer; Lung cancer (47/F)	Russian	No family history	*FANCI*	Het	Missense	NA	c.3623T>A	p.Leu1208Gln	Novel	NA	NA	NA
CRC371	Hepatic flexure of the colon cancer (50/F)	Russian	No family history	*BLM*	Het	Frameshift variant	rs1555419829	c.1316delT	p.Met439 ArgfsTer12	ClinVar	NA	NA	NA
CRC382	Rectum cancer (38/M)	Ukrainian	No family history	*BRCA2*	Het	Frameshift variant	rs886040648	c.6304_6305 delGT	p.Val2102 IlefsTer8	ClinVar	NA	NA	NA
CRC442	Rectosigmoid colon cancer (41/M)	Kazakh	No family history	*MSH6*	Het	Frameshift variant	rs55740729	c.4068_4071 dupGATT	p.Lys1358 AspfsTer2	LOVD, ClinVar	NA	NA	0.23
CRC609	Rectum cancer (40/M)	Kazakh	No family history	*APC*	Het	Frameshift variant	NA	c.907delA	p.Arg303 GlyfsTer2	Novel	NA	NA	NA
				*NBN*	Het	Frameshift variant	rs587776650	c.657_661 delACAAA	p.Lys219 AsnfsTer16	LOVD, ClinVar	NA	1.45	0.02
				*ATM, C11ORF65*	Het	Frameshift variant	NA	c.6743dupA	p.Asp2249 GlyfsTer24	Novel	NA	NA	NA
CRC380	Cancer splenic flexure (23/M)	Uigur	No family history	*MUTYH*	Het	Missense	rs34126013	c.721C>T	p.Arg241Trp	LOVD, ClinVar	NA	NA	0.01
CRC137	Sigmoid colon cancer (33/M)	Russian	No family history	*AIP*	Het	Missense	rs104894190	c.911G>A	p.Arg304Gln	ClinVar, LOVD	NA	0.07	0.14
CRC545	Rectum cancer (43/M)	Kazakh	No family history	*BRCA2*	Het	Stop-gain	rs11571833	c.9976A>T	p.Lys3326Ter	LOVD, ClinVar^#^	0.44	0.65	0.7
CRC616	Sigmoid colon cancer (17/F)	Kazakh	NA	*APC*	Het	Stop-gain	NA	c.4128T>G	p.Tyr1376Ter	Novel^#^	NA	NA	NA
CRC628	Rectum cancer (43/M)	Kazakh	No family history	*DICER1*	Het	Stop-gain	NA	c.4991C>A	p.Ser1664Ter	Novel	NA	NA	NA
CRC581	Rectum cancer (49/F)	Kazakh	No family history	*BRCA1*	Het	Splice acceptor	rs878853285	c.5341-2 delA	Unknown	ClinVar	NA	NA	NA
CRC558	Cecum cancer (37/M)	Russian	No family history	*FANCI*	Het	Splice donor	rs1556861311	c.2889+1G>A	Unknown	ClinVar	NA	NA	NA
CRC589	Rectosigmoid colon cancer (46/M)	Tatar	No family history	*BRCA2*	Het	Splice donor	rs886040935	c.6937+1G>A	Unknown	ClinVar^#^	NA	NA	NA

NA, not available; OC, ovarian cancer; BC, breast cancer; Mat.,maternal; Het, heterozygote; M, male; F, female;

# *, described in COSMIC*.

Pathogenic mutations were most common in the *APC* gene that encoded tumor suppressor protein acting as an antagonist of the Wnt signaling pathway. *APC* gene mutations were strongly associated with CRC carcinogenesis (12, 13). Most of them were found in CRC patients with polyposis, FAP, and PJS that could be considered as disease-causing mutations. Pathogenic mutations were also common in Fanconi Anemia associated gene (*FANCI*, 12.5%) known to play a significant role in repairing double-stranded DNA breaks by homologous recombination and restoring inter-chain DNA crosslinks (26). Fanconi Anemia genes have been reported to play an important role in CRC inherited pre-disposition (27). The mutations of one of the mismatch repair (MMR) genes, *MLH1* associated with Lynch syndrome (28, 29) were also quite frequent (8.3%).

Mutations were also found in non-CRC susceptibility genes such as *BRCA1* (8.3%) and *BRCA2* (12.5%). Those tumor suppressor genes controlling genome integrity were strongly associated with breast and ovarian cancer (30, 31). Pearlman et al. have shown a high prevalence of mutations in the non-CRC genes (*BRCA1/2*−8.3%) among patients with early onset of CRC (32). However, some studies have demonstrated a modest link between CRC risk and mutations in BRCA1/2 (33, 34). Notably, 1 female (Patient ID: CRC629) with a *BRCA1* mutation (c.5329dupC (also known as c.5382insC)/p.Gln1777ProfsTer74, 0.8%) with primary multiple cancer (CRC, ovarian, and breast cancer) had a family history of ovarian cancer. In the studies of CRC patients from different populations, *BRCA1* c.5382insC mutation was found in unselected Jewish Ashkenazi patients (0.44%) (25, 35), in unselected Polish patients (0.29%) and in patients with a reported family history of CRC (0.93%) (34).

### Variants of Uncertain Significance

**Figure 2:**
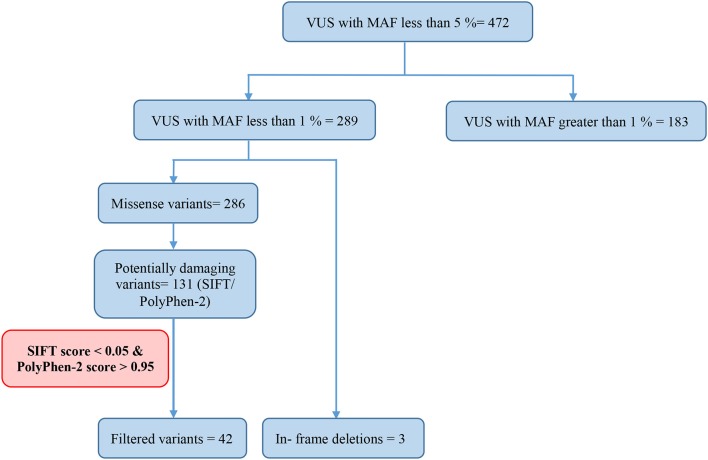
Classification of variant of uncertain significance (VUS).

**Figure 3:**
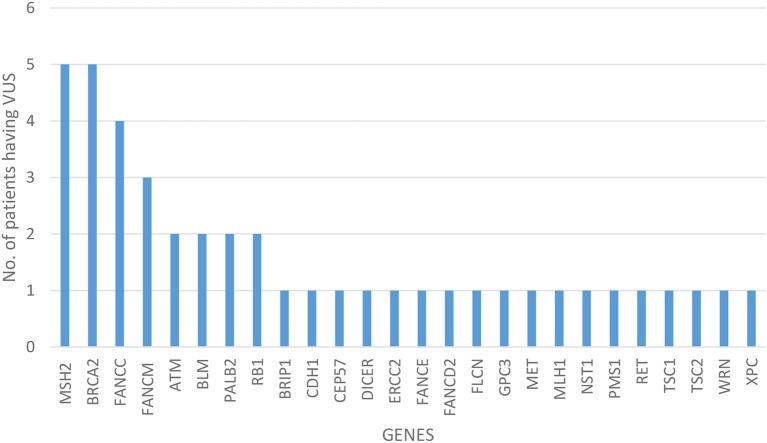
Distribution of variant of uncertain significance.

Of all the 472 identified VUS, 131 were potentially deleterious according to at least one *in silico* prediction tool (PolyPhen-2 and SIFT) (Supplementary Table 2). Forty-five of all VUS were selected according to the classification shown in Figure 2 and they were categorized as being strongly deleterious (Supplementary Table 3). Of these 45 VUS (42 missense variants in 35 patients and three in-frame deletions in three patients) were identified. Three patients (Patient ID: CRC530, CRC597, and CRC625) had in-frame deletions in the *APC, FANCE*, and *TSC2* gene, respectively. The largest number of VUSs were found in *MSH2* involved in mismatch repair and *BRCA2* which is a tumor suppressor gene involved in the maintenance of genome stability, specifically the homologous recombination pathway for double-strand DNA repair (Figure 3).

Eight novel alterations were identified. Two of the novel alterations, c.7429G>A/p.Gly2477Arg in the *ATM* gene and c.1306C > A/p.Leu436Met in the *FANCD2* gene were identified in patients with FHC (Patient ID: CRC335 and CRC587). Other six novel variants were detected in patients with no FHC, c.1135G > A/p.Ala379Thr in *NSD1* gene, c.2777A > G/p.Glu926Gly in *RB1* gene, c.2693G > A/p.Arg898Lys in the *BLM* gene, c.1031A > C/p.Gln344Pro in *MSH2* gene, c.1493T > G p.Phe498Cys in *DICER1* gene and c.278G > A/p.Arg93His in *PMS1* gene in a patient (Patient ID: CRC380, CRC442, CRC599, CRC600, and CRC635).

The three novel variants in patients (Patient ID: CRC599, CRC600, CRC635) were found in high and moderate penetrance genes (*BLM, MSH2*, and *PMS1*) which are known to be associated with CRC so these mutations can be regarded as one of the risk factors for developing CRC.

### Molecular Characteristics of Study Subgroups

**Figure 4:**
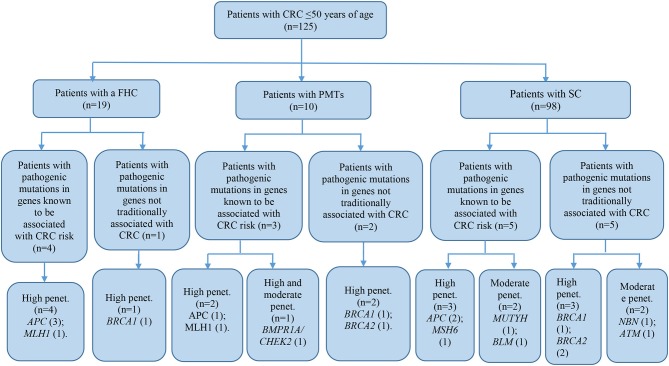
Risk assessment of pathogenic variants by the level of cancer pre-disposition genes (high and moderate) in patients with early onset of colorectal cancer. We described only patients with pathogenic mutations in genes recommended by NCCN (36, 37). CRC, colorectal cancer; FHC, family history of cancer; PMTs, primary multiple tumors; SC, sporadic cases; Penet., penetrance genes.

**Figure 5:**
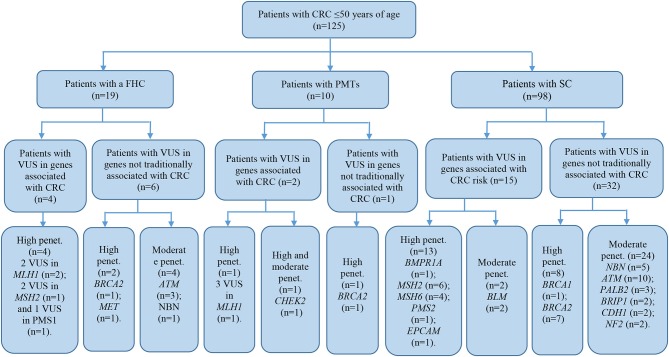
Risk assessment of VUS by the level of cancer pre-disposition genes (high and moderate) in patients with early onset of colorectal cancer. We described patients with VUS in genes recommended by NCCN (36, 37). CRC, colorectal cancer; FHC, family history of cancer; PMTs, primary multiple tumors; SC, sporadic cases; Penet., penetrance genes.

The patient cohort under study was conditionally divided into three subgroups by their clinical and molecular characteristics: patients with FHC, patients with PMTs, and patients with sporadic cases (SC) of the disease had the average age 38.9 ± 9.1, 44.6 ± 3.3, and 40.7 ± 7.3, respectively. Detailed information about the most significant mutations with deleterious effects found in patients with FHC (*n* = 19), PMTs (*n* = 10), and SCs (*n* = 98) are represented in Supplementary Tables 4–6, respectively. For patients with FHC, PMTs and SC, 32, 9, and 93 variants were classified as VUS with potentially damaging effects according to the criteria of PolyPhen and SIFT databases and six, five, and eleven variants identified were considered as pathogenic in accordance with the ACMG guidelines, respectively. Early-onset CRC patients with pathogenic variants and VUS were evaluated according to the level of cancer pre-disposition genes (high and moderate penetrance genes listed in the National Comprehensive Cancer Network (NCCN) (36, 37), which are shown in Figures 4, 5, respectively. Pathogenic mutations in high penetrance CRC genes were higher in patients with FHC (21.1 vs. 3.1%; *P* = 0.0002) and in patients with PMTs (20.0 vs. 3.1%; *P* = 0.0004) compared with patients without/unknown FHC (3.1%) (Figure 4).

### Segregation Analysis

**Figure 6:**
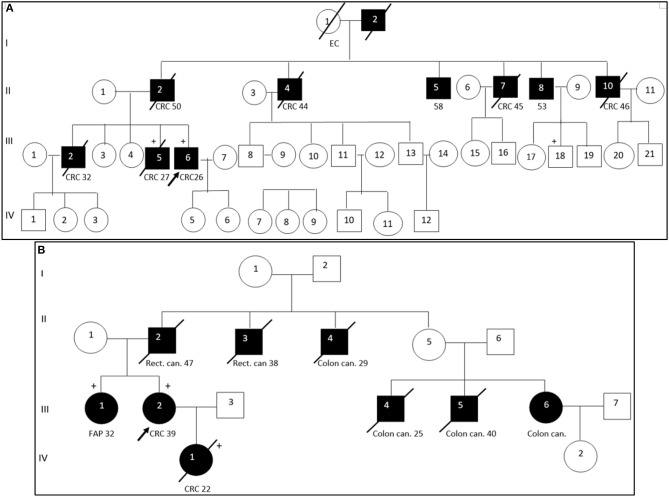
Pedigree of two patients with familial adenomatous polyposis. Arrow points to the proband. Shading indicates family members that were diagnosed with FAP. Squares and circles denote males and females, respectively. Roman number indicates generations. NGS tested patients, depicted by a plus. **(A)**. *APC* c. c.3613delA (pathogenic mutation) and *MLH1* c. c.2146G > A (VUS) were identified in III-6 (proband CRC2) and III-5 (proband's brother). *FANCI* c. c.3340delA (pathogenic mutation) were identified in III-6 (proband). **(B)**. *APC* c.4128T > G (pathogenic mutation) and *FANCM* c.4881T > G (VUS) were identified in III-2 (proband CRC613) and IV-1 (proband's daughter). *APC* c.2835delG and *MLH1* c.436C > T pathogenic mutations were identified in III-1 (proband's sister). CRC, colorectal cancer; Rect. can., rectal cancer; Colon can., colon cancer; FAP, familial adenomatous polyposis; EC, endometrial cancer.

Segregation analysis was done only for two patients with FAP and CRC with the information of relatives (Patient ID: CRC613: sister and daughter, Patient ID: CRC2 brother) but not for the whole cohort because of the unavailability of data (Figure 6). These patients had three frameshifts and two stop codon mutations in *APC, MLH1*, and *FANCI* genes. One of those mutations in the *APC* gene c.3613delA (p.Ser1205AlafsTer60) was previously described in the Polish population among family members with FAP (38). Other mutations [one in *FANCI* c.3340delA (p.Thr1114ProfsTer50), two in *APC* c.4128T > G (p.Tyr1376Ter) and c.2835delG (p.Thr946HisfsTer9), and one in *MLH1*gene c.436C > T (p.Gln146Ter)] have not been previously described in the literature. However, *APC* c.4128T > G somatic mutation has been reported in the COSMIC database.

DNA sequencing has revealed identical genetic defects in the proband's brother aged 28: germline hereditary mutations [one missense (c.2146G > A) in the *MLH1* gene and one pathogenic frameshift mutation (c.3613delA) in the *APC* gene] and daughter of a FAP (CRC613) proband with primary multiple CRC tumors had identical *APC* 4128T > G/p.Tyr1376Ter pathogenic stop-gain and *FANCM* c.4881T > G missense mutations, while the same proband's sister had another *APC* c.2835delG/p.Thr946HisfsTer9 pathogenic frameshift mutation (novel) and a *MLH1* c.436C > T/p.Gln146Ter pathogenic stop-gain mutation (described in ClinVar and LOVD) (Table 3).

## Discussion

The discovery of the molecular genetic causes of many of the hereditary syndromes associated with CRC (FAP, Lynch syndrome, Peutz-Jeghers, Gardner-Turner, Bannayan–Riley–Ruvalcaba, Bloom's disease, Tourette's syndrome, and others) has increased the importance of genetic testing (39). The limited capacity of old genetic screening methods, due to their low sensitivity and a small number of studied genes, has led to the introduction of multigenic testing in oncology (40, 41). The advantages of multigenic analysis contribute to its widespread use in clinical practice (42, 43).

The study of hereditary forms of the colon, rectal cancer and early-onset CRC in the Kazakhstani population has not been conducted previously due to the lack of statistical data. Most patients were observed at late CRC stages, and FHC was not considered. NGS-based analysis of Kazakhstani patients with early-onset CRC has been conducted for the first time. The data on the cohort of 125 patients with early CRC onset was collected in the last 5 years. According to the patient-filled questionnaire, only 19 (15.2%) patients had CRC, endometrial cancer, ovarian, breast, prostate, lung, gastric, kidney, or laryngeal cancer in their family history. Such frequency of FHC corresponds to the published data on early-onset CRC without known genetic background (44). CRC is known to be a multifactorial disease, the development of which can be influenced by lifestyle and dietary patterns (5–11). According to most of the questionnaires filled by patients with early-onset CRC, its early development can be caused by a sedentary lifestyle, smoking, as well as a diet high in red meat and low in fiber, a characteristic of traditional Kazakh cuisine.

Considering the clinical requirements in genetic testing, conducting NGS we supposed that understanding of molecular genetic variants presented in our cohort with early-onset CRC would allow segregating its hereditary forms from sporadic cases.

Using the TruSight Cancer Sequencing Panel (Illumina, USA), we have identified in total 11,152 variants in 85 genes (Supplementary Table 1). The most frequently mutated genes included: known tumor suppressor genes controlling genetic integrity (*APC, BRCA2/1*, and *ATM*), oncogenes encoding tyrosine-protein kinase transmembrane receptor activity (*RET* and *ALK*), helicase gene family (*BRIP1, WRN*, and *RECQL4*), Fanconi anemia (FA) genes (*FANCA, FANCD2*, and *FANCI*), mismatch repair endonuclease (*PMS2*) and nucleotide excision repair genes (*XPC*), nuclear tumor suppressor *MEN1* that regulates gene transcription by coordinating chromatin remodeling*, NSD1* gene that controlled the androgen receptor transactivation, *SLX4* gene that encoded an assembly component of multiple structure-specific endonucleases, and *HNF1A* gene that encoded the transcriptional factor for some specific liver enzymes responsible for detoxification. We did not find the modified variants of nine genes that were used from the panel (*CDC73, CDK4, ERCC5, MAX, SDHAF2, SDHC, SDHD, SMAD4*, and *XPA*).

Among the 289 variants of uncertain significance, 131 were attributed to potential deleterious variants. Potential deleterious VUS were obtained by mutational frequency analysis (<1% in the general population) using 1000G, ESP6500, and ExAC databases and using *in silico* predictive tools (SIFT and Polyphen2).

According to ACMG guidelines and LOVD/ClinVar databases, 24 of the altered variants were found to be pathogenic. Eight mutations were novel and were attributed to likely be pathogenic. The panel allowed the detection of the pathogenic mutations in well-established CRC susceptibility genes, such as *APC, MLH1*, and *MSH6* in high-penetrance genes, and in moderate-penetrance genes like *MUTYH* (monoallelic), *BMPR1A, BLM*, and *CHEK2* in 11 patients (8.8%) of our cohort. These findings were lower than those reported by Pearlman et al. (16%) (32). Patients with pathogenic mutations identified in CRC related and non-CRC related genes were slightly lower (16%) than the results reached by Martin-Morales et al. (18.4%) (44). In our study half of the patients with pathogenic mutations had high or moderate penetrance genes not traditionally associated with CRC (2, *BRCA1*; 3, *BRCA2*; 1, *ATM/NBN*; 3, *FANCI*; 1, *DICER1*; and 1, *AIP*). Similar results were reported in some recent studies conducted on early-onset CRC patients and unselected CRC patients of all ages (43, 45–48). The obtained data in this study cannot confirm a causal link between mutations that were detected in genes, whose significance regarding CRC pre-disposition is unknown such as breast cancer genes, Fanconi Anemia genes, etc. and developing CRC in patients. With the discovery of the germline heterozygous mutations in the study, we believe that further studies on the somatic level, such as the analysis of loss of heterozygosity, should be conducted to confirm the causative effects of mutations. Despite the fact that some studies have shown a link between CRC risk and non-CRC susceptibility gene mutations, these findings are probably incidental. However, the cancer spectrum and penetrance for many well-defined and recently discovered genes will likely redefine present multigene panel testing as using NGS is becoming more routine (32).

Dividing our study cohort into a subgroup according to the clinical features of patients with PMTs, patients with FHC, and patients with no/unknown FHC, we noticed that 50% of the pathogenic and likely pathogenic mutations were found in subgroups with FHC and PMTs. Patients with FHC and PMTs were more likely to report pathogenic mutations in high penetrance CRC genes. We assumed that those circumstances might have contributed to the active participation of those mutations in the carcinogenesis of CRC.

The syndromic nature of CRC was clinically noted in six cases (4.8%): in two cases of FAP, in three cases of LS and in one case of PJS. Mutation spectra were considered to confirm the clinical diagnosis. Thus, the patients with FAP syndrome had deleterious mutations of *APC, MLH1, FANCM*, and *FANC1* genes, while Lynch syndrome patients from our cohort had causative mutations in *MSH2, TSC1, ERCC2*, and *KIT* genes.

No pathogenic mutations were found in two patients with Lynch syndrome. However, one proband had VUS in DNA repair system genes (*MSH2* c.2078G > A (p.Cys693Tyr) and c.2072T > C (p.Ile691Thr), *TSC1* c.1460C > G (p.Ser487Cys) and *ERCC2c*.691G > A (p.Val231Met) which were considered as potentially pathogenic by two predictive analyzes *in silico*. Missense variant c.2078G > A in mismatch repair gene *MSH2* was previously described in Brazilian patients with LS (49, 50). Another mutation *MSH2* c.2072T > C/p.Ile691Thr was also reported in individuals with LS from Swiss families and in individuals from Utah and California (51, 52). This missense variant could affect the protein structure and function, but such predictions have not been verified by any functional study, and their clinical significance is still unknown. Second LS proband had no pathogenic or likely pathogenic mutations in MMR genes, but polymorphisms (*PRF1* c.272C > T/p.Ala91Val, *FANCI* c.164C > T/p.Pro55Leu, and *BRCA1* c.3113A > G/p.Glu1038Gly described in ClinVar as VUS) have been detected. The 1000G, ESP6500, and ExAC databases reported that the population frequency of those polymorphisms exceeded 1%, and two of the *in silico* predictive tools considered them as deleterious.

The pathogenic effects of two mutations (c.4128T > G and c.3613delA) in the *APC* gene were confirmed in patients with FAP (Patient ID: CRC613 and CRC2) by segregation analysis and defined as disease-causing mutations. Interestingly, the same stop-gain mutation in nucleotide position c.4128T > G (p.Tyr1376Ter) in the *APC* gene was detected in a patient with sigmoid colon cancer at the age of 17 (Patient ID: CRC616). As the patient (CRC616) had no evidence of polyposis but had the same pathogenic mutation as that of the patient aged 39 with FAP (Patient ID: CRC613), this patient was allocated to a group with a high risk of polyposis.

The patient with clinically diagnosed PJS had no mutations in the *STK11* gene which is associated with PJS (53). However, a stop-gain mutation in the *APC* gene c.3827C > G/p.Ser1276Ter was identified in that patient. Unfortunately, a segregated study was not possible because of the unavailability of the patient's relative data. We supposed possible errors in clinical diagnostics of this case. This mutation of the *APC* gene has previously been reported in Danish, Italian and Irish FAP families (54–56). Multiple pathogenic frameshift mutations in a set of genes like *APC, ATM, NBN, BMPR1A*, and *CHEK2* can be crucial in the diseases' development. Moreover, we have found multiple gene alterations with pathogenic mutations and VUS in *APC* and other MMR related genes in some cases. We assume that such alterations detected in key genes with a role in tumor suppression and mismatch repair can be the risk factor for developing polyposis and Lynch syndrome.

It should be noted, that 18 mismatch repair variants of uncertain significance (*MLH1, MSH2, MSH6, PMS1*, and *PMS2* with MAF <1% of general populations) found in 15 early-onset CRC patients could be very relevant, in particular if the tumors have high-level microsatellite instability (MSI-H) (14). However, there were no available data of the MSI instability levels for these patients.

Eleven pathogenic mutations in 11 cases were identified among 98 patients with early-onset sporadic CRC. Four of those pathogenic variants were novel. The mutation spectrum was wide and covered the genes of general replication/transcription/recombination (*BLM, DICER1*), common control of genome stability/proliferation/cell division (*BRCA1/2, APC, ATM*), DNA repair systems (*MSH6, NBN, MUTYH, FANCI*), and transcription of xenobiotic metabolizing enzymes (*AIP*). Pathogenic mutations (stop-gain, frameshift, and splice donor) were most frequent in *BRCA2, APC*, and *FANCI* genes. That subgroup of patients had many cases of VUS and polymorphisms, but a case-control study was required to assess the involvement of certain mutations in specific CRC carcinogenesis. Our previous case-control study of a panel of candidate polymorphisms has shown a significant association of the following genotypes with increased CRC risk: *DCC* (32008376 G/G(A), *MLH1* (-93G/G(A), *TP53* (Pro72Pro), *GSTT1* deletions, and *GSTM1* deletions (57). In the current study, control samples collected for NGS analysis were matched to the enrolled CRC patients by age, gender, and ethnicity to determine the role of identified variants in patients with early onset sporadic CRC.

In conclusion, we believe that despite the high cost, NGS analysis is necessary for families with a family history of CRC or CRC-related cancers to identify cause-effect mutations, clarify the clinical diagnosis and to prevent the development of disease in close relatives. The novel pathogenic mutations in high-penetrance genes obtained in our study can contribute to the explanation of CRC heritability not only in Kazakhstani families. They can supplement the list of CRC-related mutations and promote the development of cheaper specific tests for the detection of hereditary forms of CRC.

## Ethics Statement

All patients gave written informed consent in accordance with the Declaration of Helsinki. The study protocol was approved by the Ethics Committee of the Asfendiyarov Kazakh National Medical University (Almaty, Kazakhstan).

## Author Contributions

DK: supervision, funding acquisition, and project administration. SA, AP, and GZ: performed the NGS library preparation, sequencing, and bioinformatics analyses. GZ: supervised all bioinformatics analyses. GA and AJ: collected the patients' samples and clinic data. GZ and GA: writing-original draft manuscript preparation. LD and DK designed the study and contributed with the valuable discussion and revision of the manuscript. All authors read and approved the final manuscript.

### Conflict of Interest Statement

The authors declare that the research was conducted in the absence of any commercial or financial relationships that could be construed as a potential conflict of interest.

## Abbreviations

AC: Amsterdam I or II criteria
ACMG: American College of Medical Genetics and Genomics
BC: breast cancer
BG: Bethesda guidelines/criteria
BWA: Burrows-Wheeler Aligner
COSMIC: Catalog of Somatic Mutations in Cancer
CRC: colorectal cancer
ExAC: Exome Aggregation Consortium database
FAP: familial adenomatous polyposis
FDR: first-degree relative
FHC: family history of cancer
GATK: Genome Analysis Toolkit
gDNA: Genomic DNA
GWAS: genome-wide association studies
HGVS: Human Genome Variation Society
HNPCC: hereditary non-polyposis colorectal cancer
InSIGHT: International Society of Gastrointestinal Hereditary Tumors
LOVD: Leiden Open Variation Database
MAF: minor allele frequency
MMR: mismatch repair
NGS: Next-generation sequencing
OC: ovarian cancer
PJS: Peutz-Jeghers syndrome
PMTs: primary multiple tumors
SC: sporadic cases
SDR: second-degree relative
VUS: variant of uncertain significance.
