# Supplementary material for: Mutation Spectrum of Cancer-Associated Genes in Patients With Early Onset of Colorectal Cancer
Source: Front Oncol. 2019 Aug 2;9:673. doi: 10.3389/fonc.2019.00673 (PMC6688539; doi:10.3389/fonc.2019.00673)
Supplement: Supplementary file 1 [file Table_1.docx]

**Supplementary Table 1. List of 85 genes identified by TruSight Cancer Sequencing Panel.**

| **№** | **Genes** | **Gene function^a^** | **Frequency of variants (%)** |
| --- | --- | --- | --- |
| 1 | *AIP* | The protein encoded by this gene is a receptor for aryl hydrocarbons and a ligand-activated transcription factor. The encoded protein is found in the cytoplasm as part of a multiprotein complex, but upon binding of ligand is transported to the nucleus. This protein can regulate the expression of many xenobiotic metabolizing enzymes. | 222 (1.99) |
| 2 | *ALK* | This gene encodes a receptor tyrosine kinase, which belongs to the insulin receptor superfamily. This protein comprises an extracellular domain, a hydrophobic stretch corresponding to a single pass transmembrane region, and an intracellular kinase domain. It plays an important role in the development of the brain and exerts its effects on specific neurons in the nervous system. | 481 (4.31) |
| 3 | *APC* | This gene encodes a tumor suppressor protein that acts as an antagonist of the Wnt signaling pathway. It is also involved in other processes including cell migration and adhesion, transcriptional activation, and apoptosis. | 745 (6.68) |
| 4 | *ATM* | The protein encoded by this gene belongs to the PI3/PI4-kinase family. This protein is an important cell cycle checkpoint kinase that phosphorylates; thus, it functions as a regulator of a wide variety of downstream proteins, including tumor suppressor proteins p53 and BRCA1, checkpoint kinase CHK2, checkpoint proteins RAD17 and RAD9, and DNA repair protein NBS1. This protein and the closely related kinase ATR are thought to be master controllers of cell cycle checkpoint signaling pathways that are required for cell response to DNA damage and for genome stability. | 177 (1.59) |
| 5 | *BAP1* | This gene belongs to the ubiquitin C-terminal hydrolase subfamily of deubiquitinating enzymes that are involved in the removal of ubiquitin from proteins. The encoded enzyme binds to the breast cancer type 1 susceptibility protein (BRCA1) via the RING finger domain of the latter and acts as a tumor suppressor. In addition, the enzyme may be involved in regulation of transcription, regulation of cell cycle and growth, response to DNA damage and chromatin dynamics. | 1 (0.01) |
| 6 | *BLM* | The Bloom syndrome gene product is related to the RecQ subset of DExH box-containing DNA helicases and has both DNA-stimulated ATPase and ATP-dependent DNA helicase activities. Mutations causing Bloom syndrome delete or alter helicase motifs and may disable the 3'-5' helicase activity. The normal protein may act to suppress inappropriate recombination. [provided by Re, Jul 2008] | 177 (1.59) |
| 7 | *BMPR1A* | The bone morphogenetic protein (BMP) receptors are a family of transmembrane serine/threonine kinases that include the type I receptors BMPR1A and BMPR1B and the type II receptor BMPR2. These receptors are also closely related to the activin receptors, ACVR1 and ACVR2. The ligands of these receptors are members of the TGF-beta superfamily. | 74 (0.66) |
| 8 | *BRCA1* | This gene encodes a nuclear phosphoprotein that plays a role in maintaining genomic stability, and it also acts as a tumor suppressor. This protein thus plays a role in transcription, DNA repair of double-stranded breaks, and recombination. | 407 (3.65) |
| 9 | *BRCA2* | Inherited mutations in BRCA1 and this gene, BRCA2, confer increased lifetime risk of developing breast or ovarian cancer. Both BRCA1 and BRCA2 are involved in maintenance of genome stability, specifically the homologous recombination pathway for double-strand DNA repair. | 595 (5.34) |
| 10 | *BRIP1* | The protein encoded by this gene is a member of the RecQ DEAH helicase family and interacts with the BRCT repeats of breast cancer, type 1 (BRCA1). The bound complex is important in the normal double-strand break repair function of breast cancer, type 1 (BRCA1). | 303 (2.72) |
| 11 | *BUB1B* | This gene encodes a kinase involved in spindle checkpoint function. The protein has been localized to the kinetochore and plays a role in the inhibition of the anaphase-promoting complex/cyclosome (APC/C), delaying the onset of anaphase and ensuring proper chromosome segregation. Impaired spindle checkpoint function has been found in many forms of cancer. | 148 (1.33) |
| 12 | *CDH1* | This gene encodes a classical cadherin of the cadherin superfamily. Alternative splicing results in multiple transcript variants, at least one of which encodes a preproprotein that is proteolytically processed to generate the mature glycoprotein. Mutations in this gene are correlated with gastric, breast, colorectal, thyroid and ovarian cancer. | 152 (1.36) |
| 13 | *CDKN1C* | This gene is imprinted, with preferential expression of the maternal allele. The encoded protein is a tight-binding, strong inhibitor of several G1 cyclin/Cdk complexes and a negative regulator of cell proliferation. | 1 (0.01) |
| 14 | *CDKN2A* | This gene generates several transcript variants which differ in their first exons. At least three alternatively spliced variants encoding distinct proteins have been reported, two of which encode structurally related isoforms known to function as inhibitors of CDK4 kinase. | 4 (0.04) |
| 15 | *CEBPA* | This intronless gene encodes a transcription factor that contains a basic leucine zipper (bZIP) domain and recognizes the CCAAT motif in the promoters of target genes. The encoded protein functions in homodimers and also heterodimers with CCAAT/enhancer-binding proteins beta and gamma. | 1 (0.01) |
| 16 | *CEP57* | This gene encodes a cytoplasmic protein called Translokin. This protein localizes to the centrosome and has a function in microtubular stabilization. The N-terminal half of this protein is required for its centrosome localization and for its multimerization, and the C-terminal half is required for nucleating, bundling and anchoring microtubules to the centrosomes. | 81 (0.73) |
| 17 | *CHEK2* | In response to DNA damage and replication blocks, cell cycle progression is halted through the control of critical cell cycle regulators. The protein encoded by this gene is a cell cycle checkpoint regulator and putative tumor suppressor. | 138 (1.24) |
| 18 | *CYLD* | This gene is encodes a cytoplasmic protein with three cytoskeletal-associated protein-glycine-conserved (CAP-GLY) domains that functions as a deubiquitinating enzyme. Mutations in this gene have been associated with cylindromatosis, multiple familial trichoepithelioma, and Brooke-Spiegler syndrome. Alternate transcriptional splice variants, encoding different isoforms, have been characterized. | 18 (0,16) |
| 19 | *DDB2* | This gene encodes a protein that is necessary for the repair of ultraviolet light-damaged DNA. This protein is the smaller subunit of a heterodimeric protein complex that participates in nucleotide excision repair, and this complex mediates the ubiquitylation of histones H3 and H4, which facilitates the cellular response to DNA damage. | 106 (0,95) |
| 20 | *DICER1* | This gene encodes a protein possessing an RNA helicase motif containing a DEXH box in its amino terminus and an RNA motif in the carboxy terminus. The encoded protein functions as a ribonuclease and is required by the RNA interference and small temporal RNA (stRNA) pathways to produce the active small RNA component that represses gene expression. | 10 (0,09) |
| 21 | *DIS3L2* | The protein encoded by this gene is similar in sequence to 3'/5' exonucleolytic subunits of the RNA exosome. The exosome is a large multimeric ribonucleotide complex responsible for degrading various RNA substrates. Several transcript variants, some protein-coding and some not, have been found for this gene | 36 (0.32) |
| 22 | *EGFR* | The protein encoded by this gene is a transmembrane glycoprotein that is a member of the protein kinase superfamily. This protein is a receptor for members of the epidermal growth factor family. EGFR is a cell surface protein that binds to epidermal growth factor. Mutations in this gene are associated with lung cancer. | 422 (3.78) |
| 23 | *EPCAM* | This gene encodes a carcinoma-associated antigen and is a member of a family that includes at least two type I membrane proteins. This antigen is expressed on most normal epithelial cells and gastrointestinal carcinomas and functions as a homotypic calcium-independent cell adhesion molecule. | 5 (0.04) |
| 24 | *ERCC2* | The nucleotide excision repair pathway is a mechanism to repair damage to DNA. The protein encoded by this gene is involved in transcription-coupled nucleotide excision repair and is an integral member of the basal transcription factor BTF2/TFIIH complex. The gene product has ATP-dependent DNA helicase activity and belongs to the RAD3/XPD subfamily of helicases. | 85 (0.76) |
| 25 | *ERCC3* | This gene encodes an ATP-dependent DNA helicase that functions in nucleotide excision repair. The encoded protein is a subunit of basal transcription factor 2 (TFIIH) and, therefore, also functions in class II transcription. | 3 (0.03) |
| 26 | *ERCC4* | The protein encoded by this gene forms a complex with ERCC1 and is involved in the 5' incision made during nucleotide excision repair. This complex is a structure specific DNA repair endonuclease that interacts with EME1. | 68 (0.61) |
| 27 | *EXT1* | This gene encodes an endoplasmic reticulum-resident type II transmembrane glycosyltransferase involved in the chain elongation step of heparan sulfate biosynthesis. Mutations in this gene cause the type I form of multiple exostoses. [provided by RefSeq, Jul 2008] | 109 (0.98) |
| 28 | *EXT2* | This gene encodes one of two glycosyltransferases involved in the chain elongation step of heparan sulfate biosynthesis. Mutations in this gene cause the type II form of multiple exostoses. Alternatively spliced transcript variants encoding different isoforms have been noted for this gene. [provided by RefSeq, Jul 2008] | 29 (0.26) |
| 29 | *EZH2* | This gene encodes a member of the Polycomb-group (PcG) family. PcG family members form multimeric protein complexes, which are involved in maintaining the transcriptional repressive state of genes over successive cell generations. | 7 (0.06) |
| 30 | *FANCA* | The Fanconi anemia complementation group (FANC) currently includes FANCA, FANCB, FANCC, FANCD1 (also called BRCA2), FANCD2, FANCE, FANCF, FANCG, FANCI, FANCJ (also called BRIP1), FANCL, FANCM and FANCN (also called PALB2). The previously defined group FANCH is the same as FANCA. | 330 (2.96) |
| 31 | *FANCB* | This gene encodes a member of the Fanconi anemia complementation group B. This protein is assembled into a nucleoprotein complex that is involved in the repair of DNA lesions. Mutations in this gene can cause chromosome instability and VACTERL syndrome with hydrocephalus. | 11 (0,10) |
| 32 | *FANCC* | The Fanconi anemia complementation group (FANC) currently includes FANCA, FANCB, FANCC, FANCD1 (also called BRCA2), FANCD2, FANCE, FANCF, FANCG, FANCI, FANCJ (also called BRIP1), FANCL, FANCM and FANCN (also called PALB2). The previously defined group FANCH is the same as FANCA. Fanconi anemia is a genetically heterogeneous recessive disorder characterized by cytogenetic instability, hypersensitivity to DNA crosslinking agents, increased chromosomal breakage, and defective DNA repair. | 5 (0.04) |
| 33 | *FANCD2* | The Fanconi anemia complementation group (FANC) currently includes FANCA, FANCB, FANCC, FANCD1 (also called BRCA2), FANCD2, FANCE, FANCF, FANCG, FANCI, FANCJ (also called BRIP1), FANCL, FANCM and FANCN (also called PALB2). The previously defined group FANCH is the same as FANCA. The members of the Fanconi anemia complementation group do not share sequence similarity; they are related by their assembly into a common nuclear protein complex. This gene encodes the protein for complementation group D2. | 373 (3.34) |
| 34 | *FANCE* | The Fanconi anemia complementation group (FANC) currently includes FANCA, FANCB, FANCC, FANCD1 (also called BRCA2), FANCD2, FANCE, FANCF, FANCG, FANCI, FANCJ (also called BRIP1), FANCL, FANCM and FANCN (also called PALB2). | 119 (1.07) |
| 35 | *FANCF* | The Fanconi anemia complementation group (FANC) currently includes FANCA, FANCB, FANCC, FANCD1 (also called BRCA2), FANCD2, FANCE, FANCF, FANCG, FANCI, FANCJ (also called BRIP1), FANCL, FANCM and FANCN (also called PALB2). The previously defined group FANCH is the same as FANCA. Fanconi anemia is a genetically heterogeneous recessive disorder characterized by cytogenetic instability, hypersensitivity to DNA crosslinking agents, increased chromosomal breakage, and defective DNA repair. | 19 (0.17) |
| 36 | *FANCG* | The Fanconi anemia complementation group (FANC) currently includes FANCA, FANCB, FANCC, FANCD1 (also called BRCA2), FANCD2, FANCE, FANCF, FANCG, FANCI, FANCJ (also called BRIP1), FANCL, FANCM and FANCN (also called PALB2). The members of the Fanconi anemia complementation group do not share sequence similarity; they are related by their assembly into a common nuclear protein complex. This gene encodes the protein for complementation group G. | 3 (0.03) |
| 37 | *FANCI* | Plays an essential role in the repair of DNA double-strand breaks by homologous recombination and in the repair of interstrand DNA cross-links (ICLs) by promoting FANCD2 monoubiquitination by FANCL and participating in recruitment to DNA repair sites. Required for maintenance of chromosomal stability. | 266 (2.38) |
| 38 | *FANCL* | The Fanconi anemia complementation group (FANC) currently includes FANCA, FANCB, FANCC, FANCD1 (also called BRCA2), FANCD2, FANCE, FANCF, FANCG, FANCI, FANCJ (also called BRIP1), FANCL, FANCM and FANCN (also called PALB2). The previously defined group FANCH is the same as FANCA. | 112 (1.00) |
| 39 | *FANCM* | The Fanconi anemia complementation group (FANC) currently includes FANCA, FANCB, FANCC, FANCD1 (also called BRCA2), FANCD2, FANCE, FANCF, FANCG, FANCI, FANCJ (also called BRIP1), FANCL, FANCM and FANCN (also called PALB2). The previously defined group FANCH is the same as FANCA. | 150 (1.34) |
| 40 | *FH* | Fanconi anemia is a genetically heterogeneous recessive disorder characterized by cytogenetic instability, hypersensitivity to DNA crosslinking agents, increased chromosomal breakage, and defective DNA repair. The members of the Fanconi anemia complementation group do not share sequence similarity; they are related by their assembly into a common nuclear protein complex. This gene encodes the protein for complementation group M. Alternative splicing results in multiple transcript variants. | 16 (0.14) |
| 41 | *FLCN* | This gene is located within the Smith-Magenis syndrome region on chromosome 17. Mutations in this gene are associated with Birt-Hogg-Dube syndrome, which is characterized by fibrofolliculomas, renal tumors, lung cysts, and pneumothorax. Alternative splicing of this gene results in two transcript variants encoding different isoforms. | 3 (0.03) |
| 42 | *GATA2* | This gene encodes a member of the GATA family of zinc-finger transcription factors that are named for the consensus nucleotide sequence they bind in the promoter regions of target genes. The encoded protein plays an essential role in regulating transcription of genes involved in the development and proliferation of hematopoietic and endocrine cell lineages. Alternative splicing results in multiple transcript variants. | 31 (0.28) |
| 43 | *GPC3* | Cell surface heparan sulfate proteoglycans are composed of a membrane-associated protein core substituted with a variable number of heparan sulfate chains. Members of the glypican-related integral membrane proteoglycan family (GRIPS) contain a core protein anchored to the cytoplasmic membrane via a glycosyl phosphatidylinositol linkage. These proteins may play a role in the control of cell division and growth regulation. | 3 (0.03) |
| 44 | *HNF1A* | The protein encoded by this gene is a transcription factor required for the expression of several liver-specific genes. The encoded protein functions as a homodimer and binds to the inverted palindrome 5'-GTTAATNATTAAC-3'. | 280 (2.51) |
| 45 | *HRAS* | This gene belongs to the Ras oncogene family, whose members are related to the transforming genes of mammalian sarcoma retroviruses. The products encoded by these genes function in signal transduction pathways. These proteins can bind GTP and GDP, and they have intrinsic GTPase activity. This protein undergoes a continuous cycle of de- and re-palmitoylation, which regulates its rapid exchange between the plasma membrane and the Golgi apparatus. | 13 (0.12) |
| 46 | *KIT* | This gene encodes the human homolog of the proto-oncogene c-kit. C-kit was first identified as the cellular homolog of the feline sarcoma viral oncogene v-kit. This protein is a type 3 transmembrane receptor for MGF (mast cell growth factor, also known as stem cell factor). Mutations in this gene are associated with gastrointestinal stromal tumors, mast cell disease, acute myelogenous lukemia, and piebaldism. | 53 (0.47) |
| 47 | *MEN1* | This gene encodes menin, a putative tumor suppressor associated with a syndrome known as multiple endocrine neoplasia type 1. In vitro studies have shown menin is localized to the nucleus, possesses two functional nuclear localization signals, and inhibits transcriptional activation by JunD, however, the function of this protein is not known. | 249 (2.23) |
| 48 | *MET* | This gene encodes a member of the receptor tyrosine kinase family of proteins and the product of the proto-oncogene MET. The encoded preproprotein is proteolytically processed to generate alpha and beta subunits that are linked via disulfide bonds to form the mature receptor. | 157 (1.41) |
| 49 | *MLH1* | The protein encoded by this gene can heterodimerize with mismatch repair endonuclease PMS2 to form MutL alpha, part of the DNA mismatch repair system. When MutL alpha is bound by MutS beta and some accessory proteins, the PMS2 subunit of MutL alpha introduces a single-strand break near DNA mismatches, providing an entry point for exonuclease degradation. | 54 (0.48) |
| 50 | *MSH2* | This locus is frequently mutated in hereditary nonpolyposis colon cancer (HNPCC). When cloned, it was discovered to be a human homolog of the E. coli mismatch repair gene mutS, consistent with the characteristic alterations in microsatellite sequences (RER+ phenotype) found in HNPCC. | 27 (0.24) |
| 51 | *MSH6* | This gene encodes a member of the DNA mismatch repair MutS family. In E. coli, the MutS protein helps in the recognition of mismatched nucleotides prior to their repair. A highly conserved region of approximately 150 aa, called the Walker-A adenine nucleotide binding motif, exists in MutS homologs. The encoded protein heterodimerizes with MSH2 to form a mismatch recognition complex that functions as a bidirectional molecular switch that exchanges ADP and ATP as DNA mismatches are bound and dissociated. | 105 (0.94) |
| 52 | *MUTYH* | This gene encodes a DNA glycosylase involved in oxidative DNA damage repair. The enzyme excises adenine bases from the DNA backbone at sites where adenine is inappropriately paired with guanine, cytosine, or 8-oxo-7,8-dihydroguanine, a major oxidatively damaged DNA lesion. The protein is localized to the nucleus and mitochondria. | 39 (0.35) |
| 53 | *NBN* | Mutations in this gene are associated with Nijmegen breakage syndrome, an autosomal recessive chromosomal instability syndrome characterized by microcephaly, growth retardation, immunodeficiency, and cancer predisposition. The encoded protein is a member of the MRE11/RAD50 double-strand break repair complex which consists of 5 proteins. | 342 (3.07) |
| 54 | *NF1* | This gene product appears to function as a negative regulator of the ras signal transduction pathway. Mutations in this gene have been linked to neurofibromatosis type 1, juvenile myelomonocytic leukemia and Watson syndrome. The mRNA for this gene is subject to RNA editing (CGA>UGA->Arg1306Term) resulting in premature translation termination. | 165 (1.48) |
| 55 | *NF2* | This gene encodes a protein that is similar to some members of the ERM (ezrin, radixin, moesin) family of proteins that are thought to link cytoskeletal components with proteins in the cell membrane. This gene product has been shown to interact with cell-surface proteins, proteins involved in cytoskeletal dynamics and proteins involved in regulating ion transport. | 3 (0.03) |
| 56 | *NSD1* | This gene encodes a protein containing a SET domain, 2 LXXLL motifs, 3 nuclear translocation signals (NLSs), 4 plant homeodomain (PHD) finger regions, and a proline-rich region. The encoded protein enhances androgen receptor (AR) transactivation, and this enhancement can be increased further in the presence of other androgen receptor associated coregulators. | 399 (3.58) |
| 57 | *PALB2* | This gene encodes a protein that may function in tumor suppression. This protein binds to and colocalizes with the breast cancer 2 early onset protein (BRCA2) in nuclear foci and likely permits the stable intranuclear localization and accumulation of BRCA2 | 46 (0.41) |
| 58 | *PHOX2B* | The DNA-associated protein encoded by this gene is a member of the paired family of homeobox proteins localized to the nucleus. The protein functions as a transcription factor involved in the development of several major noradrenergic neuron populations and the determination of neurotransmitter phenotype. | 2 (0.02) |
| 59 | *PMS1* | This gene encodes a protein belonging to the DNA mismatch repair mutL/hexB family. This protein is thought to be involved in the repair of DNA mismatches, and it can form heterodimers with MLH1, a known DNA mismatch repair protein. Mutations in this gene cause hereditary nonpolyposis colorectal cancer type 3 (HNPCC3) either alone or in combination with mutations in other genes involved in the HNPCC phenotype, which is also known as Lynch syndrome. | 2 (0.02) |
| 60 | *PMS2* | Сomponent of the post-replicative DNA mismatch repair system (MMR). Heterodimerizes with MLH1 to form MutL alpha. DNA repair is initiated by MutS alpha (MSH2-MSH6) or MutS beta (MSH2-MSH6) binding to a dsDNA mismatch, then MutL alpha is recruited to the heteroduplex. Assembly of the MutL-MutS-heteroduplex ternary complex in presence of RFC and PCNA is sufficient to activate endonuclease activity of PMS2. | 362 (3.25) |
| 61 | *PRF1* | This gene encodes a protein with structural similarities to complement component C9 that is important in immunity. This protein forms membrane pores that allow the release of granzymes and subsequent cytolysis of target cells. Whether pore formation occurs in the plasma membrane of target cells or in an endosomal membrane inside target cells is subject to debate. | 145 (1.30) |
| 62 | *PRKAR1A* | cAMP is a signaling molecule important for a variety of cellular functions. cAMP exerts its effects by activating the cAMP-dependent protein kinase, which transduces the signal through phosphorylation of different target proteins. The inactive kinase holoenzyme is a tetramer composed of two regulatory and two catalytic subunits. | 4 (0.03) |
| 63 | *PTCH1* | This gene encodes a member of the patched family of proteins and a component of the hedgehog signaling pathway. Hedgehog signaling is important in embryonic development and tumorigenesis. The encoded protein is the receptor for the secreted hedgehog ligands, which include sonic hedgehog, Indian hedgehog and desert hedgehog. | 68 (0.61) |
| 64 | *PTEN* | This gene was identified as a tumor suppressor that is mutated in a large number of cancers at high frequency. The protein encoded by this gene is a phosphatidylinositol-3,4,5-trisphosphate 3-phosphatase. It contains a tensin like domain as well as a catalytic domain similar to that of the dual specificity protein tyrosine phosphatases. | 1 (0.01) |
| 65 | *RAD51C* | This gene is a member of the RAD51 family. RAD51 family members are highly similar to bacterial RecA and Saccharomyces cerevisiae Rad51 and are known to be involved in the homologous recombination and repair of DNA. This protein can interact with other RAD51 paralogs and is reported to be important for Holliday junction resolution. Mutations in this gene are associated with Fanconi anemia-like syndrome. | 3 (0.03) |
| 66 | *RAD51D* | The protein encoded by this gene is a member of the RAD51 protein family. RAD51 family members are highly similar to bacterial RecA and Saccharomyces cerevisiae Rad51, which are known to be involved in the homologous recombination and repair of DNA. This protein forms a complex with several other members of the RAD51 family, including RAD51L1, RAD51L2, and XRCC2. | 51 (0.46) |
| 67 | *RB1* | The protein encoded by this gene is a negative regulator of the cell cycle and was the first tumor suppressor gene found. The encoded protein also stabilizes constitutive heterochromatin to maintain the overall chromatin structure. The active, hypophosphorylated form of the protein binds transcription factor E2F1. Defects in this gene are a cause of childhood cancer retinoblastoma (RB), bladder cancer, and osteogenic sarcoma. | 5 (0.04) |
| 68 | *RECQL4* | The protein encoded by this gene is a DNA helicase that belongs to the RecQ helicase family. DNA helicases unwind double-stranded DNA into single-stranded DNAs and may modulate chromosome segregation. This gene is predominantly expressed in thymus and testis. Mutations in this gene are associated with Rothmund-Thomson, RAPADILINO and Baller-Gerold syndromes. | 466 (4.18) |
| 69 | *RET* | This gene encodes a transmembrane receptor and member of the tyrosine protein kinase family of proteins. Binding of ligands such as GDNF (glial cell-line derived neurotrophic factor) and other related proteins to the encoded receptor stimulates receptor dimerization and activation of downstream signaling pathways that play a role in cell differentiation, growth, migration and survival. | 369 (3.31) |
| 70 | *RHBDF2* | RHBDF2 (Rhomboid 5 Homolog 2) is a Protein Coding gene. Diseases associated with RHBDF2 include [Tylosis With Esophageal Cancer](http://www.malacards.org/card/tylosis_with_esophageal_cancer) and [Evans' Syndrome](http://www.malacards.org/card/evans_syndrome). Gene Ontology (GO) annotations related to this gene include *serine-type endopeptidase activity* and *growth factor binding*. An important paralog of this gene is [RHBDF1](https://www.genecards.org/cgi-bin/carddisp.pl?gene=RHBDF1). | 62 (0.55) |
| 71 | *RUNX1* | Core binding factor (CBF) is a heterodimeric transcription factor that binds to the core element of many enhancers and promoters. The protein encoded by this gene represents the alpha subunit of CBF and is thought to be involved in the development of normal hematopoiesis. | 116 (1.04) |
| 72 | *SBDS* | This gene encodes a highly conserved protein that plays an essential role in ribosome biogenesis. The encoded protein interacts with elongation factor-like GTPase 1 to disassociate eukaryotic initiation factor 6 from the late cytoplasmic pre-60S ribosomal subunit allowing assembly of the 80S subunit. Mutations within this gene are associated with the autosomal recessive disorder Shwachman-Bodian-Diamond syndrome. This gene has a closely linked pseudogene that is distally located. | 29 (0.26) |
| 73 | *SDHB* | Complex II of the respiratory chain, which is specifically involved in the oxidation of succinate, carries electrons from FADH to CoQ. The complex is composed of four nuclear-encoded subunits and is localized in the mitochondrial inner membrane. | 86 (0.77) |
| 74 | *SLX4* | This gene encodes a protein that functions as an assembly component of multiple structure-specific endonucleases. These endonuclease complexes are required for repair of specific types of DNA lesions and critical for cellular responses to replication fork failure. Mutations in this gene were found in patients with Fanconi anemia. | 354 (3.17) |
| 75 | *SMARCB1* | The protein encoded by this gene is part of a complex that relieves repressive chromatin structures, allowing the transcriptional machinery to access its targets more effectively. The encoded nuclear protein may also bind to and enhance the DNA joining activity of HIV-1 integrase. This gene has been found to be a tumor suppressor, and mutations in it have been associated with malignant rhabdoid tumors. Alternatively spliced transcript variants have been found for this gene. | 10 (0.09) |
| 76 | *STK11* | This gene, which encodes a member of the serine/threonine kinase family, regulates cell polarity and functions as a tumor suppressor. Mutations in this gene have been associated with Peutz-Jeghers syndrome, an autosomal dominant disorder characterized by the growth of polyps in the gastrointestinal tract, pigmented macules on the skin and mouth, and other neoplasms. Alternate transcriptional splice variants of this gene have been observed but have not been thoroughly characterized. | 10 (0.09) |
| 77 | *SUFU* | The Hedgehog signaling pathway plays an important role in early human development. The pathway is a signaling cascade that plays a role in pattern formation and cellular proliferation during development. This gene encodes a negative regulator of the hedgehog signaling pathway. Defects in this gene are a cause of medulloblastoma. Alternative splicing results in multiple transcript variants. | 45 (0.40) |
| 78 | *TMEM127* | This gene encodes a transmembrane protein with 3 predicted transmembrane domains. The protein is associated with a subpopulation of vesicular organelles corresponding to early endosomal structures, with the Golgi, and with lysosomes, and may participate in protein trafficking between these structures. Mutations in this gene and several other genes cause pheochromocytomas. Alternatively spliced transcript variants encoding the same protein have been identified | 5 (0.04) |
| 79 | *TP53* | This gene encodes a tumor suppressor protein containing transcriptional activation, DNA binding, and oligomerization domains. The encoded protein responds to diverse cellular stresses to regulate expression of target genes, thereby inducing cell cycle arrest, apoptosis, senescence, DNA repair, or changes in metabolism. | 59 (0.53) |
| 80 | *TSC1* | This gene is a tumor suppressor gene that encodes the growth inhibitory protein hamartin. The encoded protein interacts with and stabilizes the GTPase activating protein tuberin. This hamartin-tuberin complex negatively regulates mammalian target of rapamycin complex 1 (mTORC1) signaling which is a major regulator of anabolic cell growth. This protein also functions as a co-chaperone for Hsp90 that inhibits its ATPase activity. | 84 (0.75) |
| 81 | *TSC2* | Mutations in this gene lead to tuberous sclerosis complex. Its gene product is believed to be a tumor suppressor and is able to stimulate specific GTPases. The protein associates with hamartin in a cytosolic complex, possibly acting as a chaperone for hamartin. Alternative splicing results in multiple transcript variants encoding different isoforms. | 41 (0.37) |
| 82 | *VHL* | Von Hippel-Lindau syndrome (VHL) is a dominantly inherited familial cancer syndrome predisposing to a variety of malignant and benign tumors. A germline mutation of this gene is the basis of familial inheritance of VHL syndrome. The protein encoded by this gene is a component of the protein complex that includes elongin B, elongin C, and cullin-2, and possesses ubiquitin ligase E3 activity. | 1 (0.01) |
| 83 | *WRN* | This gene encodes a member of the RecQ subfamily of DNA helicase proteins. The encoded nuclear protein is important in the maintenance of genome stability and plays a role in DNA repair, replication, transcription and telomere maintenance. | 425 (3.81) |
| 84 | *WT1* | This gene encodes a transcription factor that contains four zinc-finger motifs at the C-terminus and a proline/glutamine-rich DNA-binding domain at the N-terminus. It has an essential role in the normal development of the urogenital system, and it is mutated in a small subset of patients with Wilms tumor. This gene exhibits complex tissue-specific and polymorphic imprinting pattern, with biallelic, and monoallelic expression from the maternal and paternal alleles in different tissues. | 43 (0.38) |
| 85 | *XPC* | The protein encoded by this gene is a key component of the XPC complex, which plays an important role in the early steps of global genome nucleotide excision repair (NER). The encoded protein is important for damage sensing and DNA binding, and shows a preference for single-stranded DNA. | 309 (2.77) |

^a^<https://www.genecards.org>

|  |
| --- |
