# Supplementary material for: Mutation Spectrum of Cancer-Associated Genes in Patients With Early Onset of Colorectal Cancer
Source: Front Oncol. 2019 Aug 2;9:673. doi: 10.3389/fonc.2019.00673 (PMC6688539; doi:10.3389/fonc.2019.00673)
Supplement: Supplementary file 2 [file Table_2.DOCX]

Supplementary Table 2. List of variants of uncertain significance which evaluated as potential damaging variants by PolyPhen-2 and SIFT

| Patient ID | Gene | Genotype | Sift | PolyPhen | HGVSc/HGVSp | dbSNP ID | 1000G | Esp6500 | ExAC | Database |
| --- | --- | --- | --- | --- | --- | --- | --- | --- | --- | --- |
| CRC2 | *MLH1* | het | tolerated (0.06) | possibly damaging (0.521) | c.2146G>A/p.Val716Met | rs35831931 | 0.04 | 0.14 | 0.12 | ClinVar/LOVD |
| CRC6 | *BRCA2* | het | deleterious (0) | Probably damaging (0.982) | c.8187G>T/p.Lys2729Asn | rs80359065 | 0.26 | NA | 0.08 | ClinVar/COSMIC |
|  | *ATM. C11ORF65* | het | tolerated (0.06) | Possibly damaging (0.459) | c.5975A>C/p.Lys1992Thr | rs150757822 | 0.02 | 0.02 | 0.04 | ClinVar/LOVD |
| CRC44 | *PALB2* | het | deleterious (0) | Probably damaging (0.997) | c.1748T>G/p.Leu583Trp | rs587782151 | NA | NA | NA | ClinVar |
| CRC82 | *EPCAM* | het | deleterious (0) | benign (0.316) | c.831A>G/p.Ile277Met | rs115283528 | 0.08 | 0.18 | 0.23 | ClinVar/LOVD |
| CRC95 | *BRCA2* | het | deleterious (0.03) | benign (0) | c.5590G>A/p.Asp1864Asn | rs587781536 | NA | NA | NA | ClinVar |
| CRC101 | *BRCA2* | het | deleterious (0.03) | benign (0) | c.5590G>A/p.Asp1864Asn | rs587781536 | NA | NA | NA | ClinVar |
| CRC137 | *VHL* | het | deleterious(0.02) | Possibly damaging (0.713) | c.74C>T/p.Pro25Leu | rs35460768 | 0.04 | 0.29 | 0.52 | ClinVar/LOVD/  COSMIC |
| CRC139 | *FANCC* | het | deleterious (0) | Probably damaging (0.998) | c.77C>T/p.Ser26Phe | rs1800361 | 0.26 | 0.5 | 0.47 | ClinVar/LOVD |
| CRC174 | *PTCH1* | het | deleterious (0.02) | Probably damaging (0.928) | c.2956G>C/p.Asp986His | NA | NA | NA | NA | Novel |
|  | *TSC1* | het | tolerated (1) | Possibly damaging (0.557) | c.819T>G/p.Asp273Glu | rs148756522 | 0.02 | 0.01 | NA | ClinVar |
| CRC205 | *NBN* | het | deleterious (0) | Possibly damaging (0.78) | c.1690G>A/p.Glu564Lys | rs72550742 | 0.24 | NA | 0.1 | ClinVar |
|  | *ATM* | het | deleterious (0) | Possibly damaging (0.797) | c.2932T>C/p.Ser978Pro | rs139552233 | 0.06 | 0.03 | 0.12 | ClinVar/LOVD/  COSMIC |
|  | *MET* | het | tolerated (0.06) | Possibly damaging (0.798) | c.3029C>T/p.Thr1010Ile | rs56391007 | 0.34 | 0.89 | 0.79 | ClinVar/COSMIC |
|  | *XPC* | het | deleterious - (0.04) | benign (0.082) | c.203A>T/p.Asp68Val | rs56012223 | 0.04 | NA | 0.03 | Novel |
| CRC238 | *RET* | het | deleterious (0.01) | Possibly damaging (0.769) | c.3112A>G/p.Thr1038Ala | rs201740483 | NA | 0.03 | 0.05 | ClinVar/LOVD/  COSMIC |
|  | *XPC* | het | tolerated (0.08) | Possibly damaging (0.536) | c.1443G>T/p.Lys481Asn | rs182616621 | 0.22 | 0.28 | 0.41 | ClinVar |
| CRC259 | *MSH2* | het | deleterious (0) | Probably damaging (1) | c.2086C>T/p.Pro696Ser | rs546201898 | NA | NA | NA | ClinVar |
| CRC283 | *FANCM* | het | deleterious (0) | Probably damaging (0.982) | c.2996C>T/p.Pro999Leu | rs148304968 | 0.08 | 0.07 | 0.03 | ClinVar/COSMIC |
|  | *PALB2* | het | tolerated (0.14) | Possibly damaging (0.595) | c.2360C>T/p.Thr787Ile | rs201042302 | 0.02 | NA | NA | ClinVar/LOVD |
| CRC330 | *MSH6* | het | tolerated (0.23) | Possibly damaging (0.458) | c.2408A>G/p.Asp803Gly | rs63751450 | 0.02 | 0.02 | 0.01 | ClinVar/LOVD |
| CRC335 | *ATM.C11ORF65* | het | deleterious (0) | Probably damaging (0.994) | c.7429G>A/p.Gly2477Arg | rs778550056 | NA | NA | NA | Novel |
| CRC368 | *FANCC* | het | tolerated (0.17) | Possibly damaging (0.467) | c.973G>A/p.Ala325Thr | rs201407189 | 0.14 | NA | 0.08 | ClinVar/LOVD/  COSMIC |
|  | *FANCA* | het | tolerated (0.24) | Possibly damaging (0.689) | c.3031C>T/p.Arg1011Cys | rs142377616 | NA | 0.01 | 0.01 | ClinVar/LOVD |
| CRC369 | *CYLD* | het | deleterious (0) | Possibly damaging (0.869) | c.806T>C/p.Met269Thr | NA | NA | NA | NA | Novel |
| CRC380 | *NSD1* | het | deleterious (0.04) | Probably damaging (0.998) | c.1135G>A/p.Ala379Thr | NA | NA | NA | NA | Novel |
|  | *PMS2* | het | deleterious (0.05) | Probably damaging (0.99) | c.751G>A/p.Val251Met | rs142434011 | NA | 0.01 | NA | ClinVar/LOVD |
|  | *FANCM* | het | deleterious (0) | Probably damaging (0.988) | c.4931G>A/p.Arg1644Gln | rs138151018 | 0.32 | 0.01 | 0.15 | ClinVar |
| CRC382 | *FLCN* | het | deleterious (0) | Probably damaging (0.999) | c.502C>T/p.Arg168Cys | rs587778367 | NA | NA | NA | ClinVar |
| CRC385 | *BRCA1* | het | tolerated (0.09) | Possibly damaging (0.691) | c.4039A>G/p.Arg1347Gly | rs28897689 | 0.06 | 0.48 | 0.4 | ClinVar/LOVD |
| CRC420 | *XPC* | het | tolerated (0.08) | Possibly damaging (0.536) | c.1443G>T/p.Lys481Asn | rs182616621 | 0.22 | 0.28 | 0.41 | ClinVar |
| CRC438 | *XPC* | het | deleterious (0) | Probably damaging (0.99) | c.872C>G/p.Ser291Cys | rs184879571 | 0.16 | 0.07 | 0.28 | ClinVar |
|  | *DICER1* | het | tolerated (0.4) | Possibly damaging (0.473) | c.2540C>G/p.Thr847Arg | NA | NA | NA | NA | Novel |
| CRC442 | *RB1* | het | deleterious(0.01) | Probably damaging (0.956) | c.2777A>G/p.Glu926Gly | NA | NA | NA | NA | Novel |
| CRC457 | *BMPR1A* | het | tolerated (0.07) | Probably damaging (0.97) | c.316T>C/p.Ser106Pro | NA | NA | NA | NA | Novel |
| CRC520 | *FANCL* | het | deleterious (0.02) | benign (0.099) | c.108C>G/p.Phe36Leu | rs149726602 | 0.18 | 0.02 | 0.13 | ClinVar/COSMIC |
| CRC526 | *NBN* | het | deleterious (0.02) | benign (0.163) | c.511A>G/p.Ile171Val | rs61754966 | 0.04 | 0.12 | 0.14 | ClinVar/LOVD |
| CRC529 | *FANCA* | het | tolerated (0.24) | Possibly damaging (0.689) | c.3031C>T/p.Arg1011Cys | rs142377616 | NA | 0.01 | 0.01 | ClinVar/LOVD |
| CRC530 | *WRN* | het | deleterious (0.01) | Possibly damaging (0.792) | c.95A>G/p.Lys32Arg | rs34477820 | 0.12 | 0.3 | 0.37 | ClinVar/LOVD |
| CRC544 | *WRN* | het | deleterious (0.01) | Probably damaging (0.997) | c.2059T>G/p.Leu687Val | rs185468906 | 0.08 | NA | 0.1 | ClinVar |
|  | *RET* | het | tolerated - lowconfidence (0.13) | Possibly damaging (0.841) | c.129C>A/p.Asp43Glu |  | NA | NA | NA | Novel |
| CRC546 | *ATM* | het | deleterious (0.05) | Possibly damaging (0.53) | c.1810C>T/p.Pro604Ser | rs2227922 | 0.26 | 0.4 | 0.31 | ClinVar/LOVD/  COSMIC |
|  | *ATM* | het | deleterious (0) | Probably damaging (0.984) | c.4388T>G/p.Phe1463Cys | rs138327406 | 0.04 | 0.11 | 0.14 | ClinVar/COSMIC |
| CRC547 | *PRF1* | het | deleterious (0.03) | benign (0.006) | c.755A>G/p.Asn252Ser | rs28933375 | 0.76 | 0.85 | 0.52 | ClinVar/LOVD/  COSMIC |
| CRC548 | *XPC* | het | tolerated (0.06) | Probably damaging (0.945) | c.923C>T/p.Ala308Val | NA | NA | NA | NA | Novel |
| CRC551 | *MET* | het | deleterious (0.04) | Probably damaging (0.985) | c.632T>G/p.Leu211Trp | rs45483396 | 0.04 | NA | 0.03 | ClinVar |
|  | *NBN* | het | deleterious (0) | Possibly damaging (0.78) | c.1690G>A/p.Glu564Lys | rs72550742 | 0.24 | NA | 0.1 | ClinVar/LOVD |
|  | *BRIP1* | het | deleterious (0) | Probably damaging (0.996) | c.1902G>C/p.Gln634His | rs1060501748 | NA | NA | NA | ClinVar |
| CRC553 | *BRCA2* | het | deleterious (0) | Probably damaging (0.998) | c.10045A>G/p.Thr3349Ala | rs80358387 | NA | 0.02 | NA | ClinVar/LOVD |
|  | *CHEK2* | het | deleterious (0) | Probably damaging (0.913) | c.308T>C/p.Phe103Ser | NA | NA | NA | NA | ClinVar |
|  | *FANCA* | het | deleterious (0) | benign (0.152) | c.1874G>C/p.Cys625Ser | rs139235751 | 0.12 | 0.23 | 0.28 | ClinVar |
| CRC558 | *DICER1* | het | tolerated (0.3) | Possibly damaging (0.844) | c.3295A>G/p.Lys1099Glu | NA | NA | NA | NA | ClinVar |
| CRC569 | *FANCA* | het | deleterious (0.02) | Possibly damaging (0.587) | c.184C>T/p.Leu62Phe | NA | NA | NA | NA | Novel |
| CRC570 | *FANCC* | het | deleterious (0) | Probably damaging (0.997) | c.584A>T/p.Asp195Val | rs1800365 | 0.26 | 0.31 | 0.31 | ClinVar/LOVD |
| CRC573 | *MET* | het | tolerated (0.06) | Possibly damaging (0.798) | c.3029C>T/p.Thr1010Ile | rs56391007 | 0.34 | 0.89 | 0.79 | ClinVar/COSMIC |
|  | *RET* | het | deleterious (0.02) | benign (0.055) | c.1717G>A/p.Val573Met | rs758766818 | NA | NA | NA | Novel/COSMIC |
| CRC579 | *PALB2* | het | deleterious (0) | Probably damaging (0.971) | c.3296C>G/p.Thr1099Arg | rs142132127 | 0.02 | NA | 0.01 | ClinVar/COSMIC |
| CRC580 | *NBN* | het | deleterious (0.02) | benign (0.163) | c.511A>G/p.Ile171Val | rs61754966 | 0.04 | 0.12 | 0.14 | ClinVar/LOVD |
| CRC581 | *NF2* | het | tolerated (0.13) | Possibly damaging (0.553) | c.1439C>T/p.Thr480Met | rs145666157 | 0.04 | NA | 0.04 | ClinVar/COSMIC |
| CRC584 | *NSD1* | het | deleterious (0) | Possibly damaging (0.513) | c.3133C>T/p.Arg1045Cys | rs377148087 | NA | 0.01 | NA | ClinVar |
|  | *TSC2* | het | deleterious (0.01) | benign (0.209) | c.3475C>T/p.Arg1159Trp | rs45517295 | 0.16 | 0 | 0.04 | ClinVar |
| CRC585 | *FANCD2* | het | tolerated (0.1) | Possibly damaging (0.605) | c.78A>C/p.Gln26His | rs45510294 | 0.02 | 0.06 | 0.06 | ClinVar/LOVD |
| CRC586 | *CDH1* | het | deleterious (0) | Probably damaging (1) | c.2494G>A/p.Val832Met | rs35572355 | 0.04 | 0.02 | 0.02 | ClinVar |
| CRC587 | *FANCD2* | het | deleterious (0) | Probably damaging (0.97) | c.1306C>A/p.Leu436Met | rs373898927 | NA | 0.01 | NA | Novel/COSMIC |
|  | *PMS1* | het | deleterious (0.01) | benign (0.375) | c.2780A>G/p.Tyr927Cys | rs111254723 | NA | NA | NA | Novel |
| CRC589 | *MSH6* | het | deleterious (0) | Probably damaging (0.928) | c.3488A>T/p.Glu1163Val | rs63750252 | 0.28 | NA | 0.12 | ClinVar/LOVD/  COSMIC |
|  | *FANCC* | het | deleterious (0) | Probably damaging (0.998) | c.77C>T/p.Ser26Phe | rs1800361 | 0.26 | 0.5 | 0.47 | ClinVar/LOVD |
| CRC592 | *BLM* | het | deleterious (0) | Probably damaging (0.99) | c.2293G>A/p.Val765Ile | rs191789336 | 0.02 | NA | 0.03 | ClinVar/LOVD |
| CRC593 | *PRKAR1A* | het | deleterious (0) | Possibly damaging (0.805) | c.287G>T/p.Arg96Leu | NA | NA | NA | NA | Novel |
| CRC594 | *MLH1* | het | deleterious (0) | Probably damaging (0.994) | c.649C>T/p.Arg217Cys | rs4986984 | 0.06 | NA | 0.03 | ClinVar/COSMIC |
| CRC599 | *BLM* | het | deleterious (0) | Probably damaging (0.998) | c.2693G>A/p.Arg898Lys | NA | NA | NA | NA | Novel |
|  | *ATM* | het | deleterious (0) | benign (0.137) | c.5558A>T/p.Asp1853Val | rs1801673 | 0.18 | 0.48 | 0.52 | ClinVar/LOVD/  COSMIC |
|  | *DICER1* | het | deleterious (0.01) | benign (0.006) | c.484G>T/p.Gly162Cys |  | NA | NA | NA | Novel |
| CRC600 | *MSH2* | het | deleterious (0) | Probably damaging (0.974) | c.1031A>C/p.Gln344Pro | NA | NA | NA | NA | Novel |
|  | *DICER1* | het | deleterious (0.04) | Probably damaging (0.998) | c.1493T>G/p.Phe498Cys | NA | NA | NA | NA | Novel |
| CRC601 | *RET* | het | deleterious (0.01) | Probably damaging (0.996) | c.874G>A/p.Val292Met | rs34682185 | 0.38 | NA | 0.05 | ClinVar |
|  | *FANCM* | het | deleterious (0) | Probably damaging (0.988) | c.4931G>A/p.Arg1644Gln | rs138151018 | 0.32 | 0.01 | 0.15 | ClinVar |
|  | *MSH2* | het | deleterious (0.01) | benign (0.247) | c.1168C>T/p.Leu390Phe | rs17224367 | 0.28 | 0.01 | 0.16 | ClinVar/LOVD |
| CRC602 | *BRCA2* | het | deleterious (0.01) | Possibly damaging (0.875) | c.5070A>C/p.Lys1690Asn | rs56087561 | NA | 0.02 | 0.02 | ClinVar/LOVD |
|  | *NSD1* | het | tolerated - (0.3) | Possibly damaging (0.724) | c.1865G>C/p.Cys622Ser | NA | NA | NA | NA | Novel |
| CRC603 | *MSH6* | het | tolerated (0.24) | Probably damaging (0.998) | c.1481C>T/p.Ala494Val | NA | NA | NA | NA | ClinVar |
|  | *ATM* | het | deleterious (0) | benign (0.137) | c.5558A>T/p.Asp1853Val | rs1801673 | 0.18 | 0.48 | 0.52 | ClinVar/LOVD/  COSMIC |
| CRC605 | *MSH2* | het | deleterious (0) | Probably damaging (0.998) | c.1882G>C/p.Gly628Arg | rs371776176 | NA | NA | NA | ClinVar |
|  | *ERCC4* | het | deleterious (0.01) | Possibly damaging (0.448) | c.2734G>A/p.Gly912Arg | rs150077735 | 0.02 | NA | 0.02 | ClinVar |
|  | *CDH1* | het | deleterious (0) | Probably damaging (1) | c.2494G>A/p.Val832Met | rs35572355 | 0.04 | 0.02 | 0.02 | ClinVar |
| CRC606 | *MSH6* | het | deleterious (0) | Probably damaging (0.928) | c.3488A>T/p.Glu1163Val | rs63750252 | 0.28 | NA | 0.12 | ClinVar/LOVD/  COSMIC |
|  | *MSH2* | het | deleterious (0.01) | benign (0.247) | c.1168C>T/p.Leu390Phe | rs17224367 | 0.28 | 0.01 | 0.16 | ClinVar/LOVD |
|  | *NBN* | het | deleterious (0.02) | benign (0.163) | c.511A>G/p.Ile171Val | rs61754966 | 0.04 | 0.12 | 0.14 | ClinVar/LOVD |
| CRC607 | *MSH2* | het | deleterious (0) | Probably damaging (0.992) | c.2078G>A/p.Cys693Tyr | rs1057524909 | NA | NA | NA | ClinVar/LOVD |
|  | *TSC1* | het | deleterious (0.02) | Probably damaging (0.999) | c.1460C>G/p.Ser487Cys | rs118203532 | 0.02 | 0.02 | 0.04 | ClinVar |
|  | *ERCC2* | het | deleterious (0) | Probably damaging (0.983) | c.691G>A/p.Val231Met | rs200895828 | 0.06 | NA | 0.01 | ClinVar/COSMIC |
|  | *MSH2* | het | tolerated (0.07) | Probably damaging (0.995) | c.2072T>C/p.Ile691Thr | rs754824872 | NA | NA | NA | ClinVar |
|  | *KIT* | het | deleterious (0) | benign (0.259) | c.1352C>T/p.Ser451Phe | NA | NA | NA | NA | ClinVar/COSMIC |
| CRC609 | *MSH2* | het | deleterious (0.02) | Probably damaging (0.984) | c.2542G>T/p.Ala848Ser | rs746972142 | NA | NA | NA | ClinVar |
|  | *ATM* | het | deleterious (0.02) | benign (0.15) | c.4612G>C/p.Val1538Leu | NA | NA | NA | NA | ClinVar |
| CRC610 | *BRIP1* | het | tolerated (0.12) | Possibly damaging (0.856) | c.430G>A/p.Ala144Thr | rs116952709 | 0.46 | 0.02 | 0.13 | ClinVar/COSMIC |
| CRC611 | *BRCA2* | het | deleterious (0) | Probably damaging (0.979) | c.7544C>T/p.Thr2515Ile | rs28897744 | NA | 0.05 | 0.07 | ClinVar/LOVD |
| CRC612 | *FANCC* | het | deleterious (0) | Probably damaging (0.998) | c.77C>T/p.Ser26Phe | rs1800361 | 0.26 | 0.5 | 0.47 | ClinVar/LOVD |
| CRC613 | *MLH1* | het | deleterious (0) | Possibly damaging (0.714) | c.1852A>G/p.Lys618Glu | rs35001569 | 0.32 | 0.37 | 0.34 | ClinVar/LOVD/COSMIC |
|  | *FANCM* | het | deleterious (0.01) | Possibly damaging (0.556) | c.4881T>G/p.Phe1627Leu | NA | NA | NA | NA | LOVD |
| CRC618 | *RB1* | het | deleterious (0) | Probably damaging (0.996) | c.2392C>T/p.Arg798Trp | rs187912365 | 0.08 | 0.01 | 0.01 | ClinVar/COSMIC |
| CRC620 | *BRCA2* | het | deleterious (0.01) | Possibly damaging (0.773) | c.3349A>G/p.Ile1117Val | rs397507307 | NA | NA | NA | ClinVar/LOVD |
|  | *DICER1* | het | deleterious (0.02) | Possibly damaging (0.746) | c.1381A>G/p.Ile461Val | rs141163928 | NA | 0.02 | 0.01 | ClinVar |
| CRC621 | *NBN* | het | deleterious (0.02) | Possibly damaging (0.558) | c.1670C>A/p.Ala557Asp | NA | NA | NA | NA | Novel |
|  | *FANCI* | het | tolerated (0.06) | Possibly damaging (0.86) | c.1813C>T/p.Leu605Phe | rs117125761 | 0.2 | 0.76 | 0.62 | ClinVar/LOVD |
|  | *NSD1* | het | deleterious (0.02) | benign (0.002) | c.2456C>A/p.Thr819Asn | NA | NA | NA | NA | Novel |
| CRC622 | *GPC3* | hom | deleterious (0.02) | Probably damaging (0.978) | c.1354G>A/p.Val452Met | rs11539789 | 0.45 | 0.27 | 0.58 | ClinVar/LOVD |
| CRC623 | *WRN* | het | deleterious (0) | Possibly damaging (0.631) | c.2983G>A/p.Ala995Thr | rs140768346 | 0.08 | 0.21 | 0.22 | ClinVar |
| CRC624 | *CEP57* | het | deleterious (0.01) | Probably damaging (0.998) | c.333G>C/p.Gln111His | rs117321017 | 0.4 | 0.58 | 0.59 | ClinVar/LOVD |
| CRC625 | *MET* | het | tolerated (0.06) | Possibly damaging (0.798) | c.3029C>T/p.Thr1010Ile | rs56391007 | 0.34 | 0.89 | 0.79 | ClinVar/COSMIC |
| CRC626 | *ATM* | het | deleterious (0) | Possibly damaging (0.841) | c.146C>G/p.Ser49Cys | rs1800054 | 0.42 | 0.99 | 0.74 | ClinVar/LOVD |
|  | *FANCA* | het | deleterious (0) | Possibly damaging (0.759) | c.1592A>G/p.Tyr531Cys | NA | NA | NA | NA | Novel |
| CRC627 | *SLX4* | het | tolerated (0.21) | Probably damaging (0.999) | c.1442G>A/p.Arg481Gln | rs145194745 | 0.02 | 0.01 | NA | Novel |
|  | *ATM* | het | deleterious (0) | benign (0.22) | c.1229T>C/p.Val410Ala | rs56128736 | 0.08 | 0.18 | 0.22 | Novel/COSMIC |
| CRC628 | *BRCA2* | het | deleterious (0.03) | benign (0.002) | c.6100C>T/p.Arg2034Cys | rs1799954 | 0.14 | 0.4 | 0.32 | ClinVar/LOVD/  COSMIC |
| CRC629 | *APC* | het |  | Probably damaging (0.997) | c.6724A>G/p.Ser2242Gly | rs201375478 | NA | 0.01 | 0.01 | ClinVar/LOVD |
|  | *CEP57* | het | deleterious (0) | Possibly damaging (0.882) | c.949C>T/p.His317Tyr | rs140320103 | 0.02 | 0.05 | 0.01 | ClinVar |
| CRC630 | *AIP* | het | deleterious (0.05) | Possibly damaging (0.459) | c.47G>A/p.Arg16His | rs145047094 | 0.06 | 0.25 | 0.2 | ClinVar/LOVD |
| CRC632 | *WRN* | het | deleterious (0) | Possibly damaging (0.631) | c.2983G>A/p.Ala995Thr | rs140768346 | 0.08 | 0.21 | 0.22 | ClinVar |
|  | *FANCI* | het | tolerated (0.06) | Possibly damaging (0.86) | c.1813C>T/p.Leu605Phe | rs117125761 | 0.2 | 0.76 | 0.62 | ClinVar/LOVD |
| CRC635 | *PMS1* | het | deleterious (0) | Probably damaging (0.98) | c.278G>A/p.Arg93His | rs778185859 | NA | NA | NA | Novel |
|  | *RET* | het | deleterious (0) | Possibly damaging (0.857) | c.937C>T/p.Arg313Trp | NA | NA | NA | NA | ClinVar |
|  | *ATM.C11ORF65* | het | tolerated (0.08) | Possibly damaging (0.53) | c.7235A>G/p.Asn2412Ser | rs786203311 | NA | NA | NA | ClinVar |
| CRC638 | *XPC* | het | deleterious (0.01) | Possibly damaging (0.61) | c.281G>T/p.Ser94Ile | NA | NA | NA | NA | Novel |
|  | *ATM* | het | deleterious (0.02) | Probably damaging (0.95) | c.107A>G/p.Asp36Gly | NA | NA | NA | NA | ClinVar/LOVD |
|  | *NSD1* | het | deleterious (0.01) | benign (0.013) | c.7852G>A/p.Val2618Ile | rs373787813 | 0.02 | 0.02 | 0.01 | Novel |
| CRC639 | *WRN* | het | deleterious (0.01) | Possibly damaging (0.792) | c.95A>G/p.Lys32Arg | rs34477820 | 0.12 | 0.3 | 0.37 | ClinVar/LOVD |
|  | *BRCA2* | het | deleterious (0) | Probably damaging (0.979) | c.7544C>T/p.Thr2515Ile | rs28897744 | 0 | 0.05 | 0.07 | ClinVar/LOVD |
| CRC640 | *TSC2* | het | deleterious (0) | Possibly damaging (0.707) | c.275A>T/p.Glu92Val | rs137853994 | 0.06 | 0.15 | 0.19 | ClinVar/LOVD |
| CRC641 | *NF2* | het | tolerated (0.13) | Possibly damaging (0.553) | c.1439C>T/p.Thr480Met | rs145666157 | 0.04 | 0 | 0.04 | ClinVar/COSMIC |

Abbreviations: NA – not available; Het – heterozygote.
