# Supplementary material for: Mutation Spectrum of Cancer-Associated Genes in Patients With Early Onset of Colorectal Cancer
Source: Front Oncol. 2019 Aug 2;9:673. doi: 10.3389/fonc.2019.00673 (PMC6688539; doi:10.3389/fonc.2019.00673)
Supplement: Supplementary file 3 [file Table_3.DOCX]

Supplementary Table 3. Variants of uncertain significance strongly suspected of being deleterious mutations

| Patient ID | Clinical features (age at diagnosis/gender) | Ethnicity | Family history | Gene | Sift | Poly  Phen | HGVSc | HGVSp | Consequenceand dbSNP ID | Database | Population frequency | | |
| --- | --- | --- | --- | --- | --- | --- | --- | --- | --- | --- | --- | --- | --- |
|  |  |  |  |  |  |  |  |  |  |  | 1000G | Esp6500 | ExAC |
| CRC6 | Rectosigmoid colon cancer (39/F) | Kazakh | Non | *BRCA2* | 0 | 0.982 | c.8187G>T | p.Lys2729Asn | missense rs80359065 | ClinVar | 0.26 | NA | 0.08 |
| CRC139 | Primary multiple synchronous cancer. Rectosigmoid colon cancer; Cancer splenic flexure; OC (46/F) | Russian | Non | *FANCC* | 0 | 0.998 | c.77C>T | p.Ser26Phe | missense rs1800361 | ClinVar | 0.26 | 0.5 | 0.47 |
| CRC259 | Cecum cancer (46/M) | Kazakh | Non | *MSH2* | 0 | 1 | c.2086C>T | p.Pro696Ser | missense rs546201898 | ClinVar | NA | NA | NA |
| CRC283 | Rectum cancer (29/F) | Kazakh | Non | *FANCM* | 0 | 0.982 | c.2996C>T | p.Pro999Leu | missense rs148304968 | ClinVar | 0.08 | 0.07 | 0.03 |
| CRC44 | Rectum cancer (49/F) | Kazakh | Non | *PALB2* | 0 | 0.997 | c.1748T>G | p.Leu583Trp | missense rs587782151 | ClinVar | NA | NA | NA |
| CRC335 | Rectosigmoid colon cancer (47/F) | Russian | father: prostate cancer | *ATM* | 0 | 0.994 | c.7429G>A | p.Gly2477Arg | missense rs778550056 | Novel | NA | NA | NA |
| CRC380 | Cancer splenic flexure (23/M) | Uigur | Non | *NSD1* | 0.04 | 0.998 | c.1135G>A | p.Ala379Thr | NA | Novel | NA | NA | NA |
|  |  |  |  | *FANCM* | 0 | 0.988 | c.4931G>A | p.Arg1644Gln | missense rs138151018 | ClinVar | 0.32 | 0.01 | 0.15 |
| CRC382 | Primary multiple synchronous cancer. Rectum cancer; Cancer of both adrenal glands (38/M) | Ukrainian | Non | *FLCN* | 0 | 0.999 | c.502C>T | p.Arg168Cys | missense rs587778367 | ClinVar | NA | NA | NA |
| CRC438 | Rectosigmoid colon cancer (49/F) | Kazakh | Non | *XPC* | 0 | 0.99 | c.872C>G | p.Ser291Cys | missense rs184879571 | ClinVar | 0.16 | 0.07 | 0.28 |
| CRC442 | Rectosigmoid colon cancer (41/M) | Kazakh |  | *RB1* | 0.01 | 0.956 | c.2777A>G | p.Glu926Gly | NA | Novel | NA | NA | NA |
| CRC544 | Sigmoid colon cancer (50/F) | Russian | Non | *WRN* | 0.01 | 0.997 | c.2059T>G | p.Leu687Val | missense rs185468906 | ClinVar | 0.08 | NA | 0.1 |
| CRC546 | Sigmoid colon cancer (38/F) | Father:Uzbek; Mother: Kazakh | Non | *ATM* | 0 | 0.984 | c.4388T>G | p.Phe1463Cys | missense rs138327406 | ClinVar | 0.04 | 0.11 | 0.14 |
| CRC551 | Sigmoid colon cancer (50/M) | Kazakh | Non | *MET* | 0.04 | 0.985 | c.632T>G | p.Leu211Trp | missense rs45483396 | ClinVar | 0.04 | NA | 0.03 |
|  |  |  |  | *BRIP1* | 0 | 0.996 | c.1902G>C | p.Gln634His | missense rs1060501748 | ClinVar | NA | NA | NA |
| CRC553 | Primary multiple metachronous cancer. Sigmoid colon cancer; Lung cancer (47/F) | Russian | Non | *BRCA2* | 0 | 0.998 | c.10045A>G | p.Thr3349Ala | missense rs80358387 | ClinVar/LOVD | NA | 0.02 | NA |
| CRC570 | Primary multiple metachronous cancer. Gastric cancer; Rectosigmoid colon cancer (46/F) | Kazakh | Non | *FANCC* | 0 | 0.997 | c.584A>T | p.Asp195Val | missense rs1800365 | ClinVar/LOVD | 0.26 | 0.31 | 0.31 |
| CRC579 | Rectum cancer (45/M) | Uigur | Non | *PALB2* | 0 | 0.971 | c.3296C>G | p.Thr1099Arg | missense rs142132127 | ClinVar | 0.02 | NA | 0.01 |
| CRC586 | Sigmoid colon cancer (37/M) | Kazakh | Non | *CDH1* | 0 | 1 | c.2494G>A | p.Val832Met | missense rs35572355 | ClinVar | 0.04 | 0.02 | 0.02 |
| CRC587 | Rectum cancer (49/M) | Kazakh | father: kidney cancer | *FANCD2* | 0 | 0.97 | c.1306C>A | p.Leu436Met | missense rs373898927 | Novel | NA | 0.01 | NA |
| CRC589 | Rectosigmoid colon cancer (46/M) | Tatar | Non | *FANCC* | 0 | 0.998 | c.77C>T | p.Ser26Phe | missense rs1800361 | ClinVar/LOVD | 0.26 | 0.5 | 0.47 |
| CRC592 | Sigmoid colon cancer (40/M) | Korean | Non | *BLM* | 0 | 0.99 | c.2293G>A | p.Val765Ile | missense rs191789336 | ClinVar | 0.02 | NA | 0.03 |
| CRC594 | Rectum cancer (42/F) | Kazakh | Non | *MLH1* | 0 | 0.994 | c.649C>T | p.Arg217Cys | missense rs4986984 | ClinVar | 0.06 | NA | 0.03 |
| CRC599 | Sigmoid colon cancer (32/F) | Kazakh | Non | *BLM* | 0 | 0.998 | c.2693G>A | p.Arg898Lys | NA | Novel | NA | NA | NA |
| CRC600 | Rectum cancer (45/M) | Kazakh | Non | *MSH2* | 0 | 0.974 | c.1031A>C | p.Gln344Pro | NA | Novel | NA | NA | NA |
|  |  |  |  | *DICER1* | 0.04 | 0.998 | c.1493T>G | p.Phe498Cys | NA | Novel | NA | NA | NA |
| CRC601 | Rectum cancer (38/M) | Dungan | Non | *RET* | 0.01 | 0.996 | c.874G>A | p.Val292Met | missense rs34682185 | ClinVar | 0.38 | NA | 0.05 |
|  |  |  |  | *FANCM* | 0 | 0.988 | c.4931G>A | p.Arg1644Gln | missense rs138151018 | ClinVar | 0.32 | 0.01 | 0.15 |
| CRC605 | Sigmoid colon cancer (45/M) | Uigur | Non | *MSH2* | 0 | 0.998 | c.1882G>C | p.Gly628Arg | missense rs371776176 | ClinVar | NA | NA | NA |
|  |  |  |  | *CDH1* | 0 | 1 | c.2494G>A | p.Val832Met | missense rs35572355 | ClinVar | 0.04 | 0.02 | 0.02 |
| CRC607 | Ascending colon cancer (18/M) | Kazakh | Mat.great-grandmother: endometrial cancer;  Mat. grandmother: gastric cancer; Mat. Aunt (sister of mother): CRC; One aunt of mother: gastric cancer;  Other aunt of mother: CRC. | *MSH2* | 0 | 0.992 | c.2078G>A | p.Cys693Tyr | missense rs1057524909 | ClinVar/LOVD | NA | NA | NA |
|  |  |  |  | *TSC1* | 0.02 | 0.999 | c.1460C>G | p.Ser487Cys | missense rs118203532 | ClinVar | 0.02 | 0.02 | 0.04 |
|  |  |  |  | *ERCC2* | 0 | 0.983 | c.691G>A | p.Val231Met | missense rs200895828 | ClinVar | 0.06 | NA | 0.01 |
| CRC609 | Rectum cancer (40/M) | Kazakh | Non | *MSH2* | 0.02 | 0.984 | с.2542G>T | p.Ala848Ser | missense rs746972142 | ClinVar | NA | NA | NA |
| CRC611 | Rectum cancer (43/M) | Kazakh | Non | *BRCA2* | 0 | 0.979 | c.7544C>T | p.Thr2515Ile | missense rs28897744 | ClinVar/LOVD | NA | 0.05 | 0.07 |
| CRC612 | Rectosigmoid colon cancer (38/F) | Russian | Non | *FANCC* | 0 | 0.998 | c.77C>T | p.Ser26Phe | missense rs1800361 | ClinVar | 0.26 | 0.5 | 0.47 |
| CRC613 | FAP; Primary multiple synchronous cancer. Cancer splenic flexure; Rectum Cancer (39/F) | Russian | Family member with FAP and CRC | *MLH1* | 0 | 0.955 | c.1853A>C | p.Lys618Thr | missense rs63750449 | ClinVar/LOVD | 0.32 | 0.38 | 0.34 |
| CRC618 | Rectum cancer (47/F) | Russian | Non | *RB1* | 0 | 0.996 | c.2392C>T | p.Arg798Trp | missense rs187912365 | ClinVar | 0.08 | 0.01 | 0.01 |
| CRC622 | Cecum cancer (43/M) | Russian | mother: ascending colon cancer; mat. mother: gastric cancer | *GPC3* | 0.02 | 0.978 | c.1354G>A | p.Val452Met | missense rs11539789 | ClinVar | 0.45 | 0.27 | 0.58 |
| CRC624 | Primary multiple synchronous cancer. Sigmoid colon cancer; Hepatic flexure of the colon cancer (46/M) | Russian | Non | *CEP57* | 0.01 | 0.998 | c.333G>C | p.Gln111His | missense rs117321017 | ClinVar/LOVD | 0.4 | 0.58 | 0.59 |
| CRC635 | Hepatic flexure of the colon cancer (37/M) | Father: Bashkir; Mother: Kazakh | Non | *PMS1* | 0 | 0.98 | c.278G>A | p.Arg93His | missense rs778185859 | Novel | NA | NA | NA |
| CRC639 | Sigmoid colon cancer (49/F) | Russian | Mother: Rectum cancer | *BRCA2* | 0 | 0.979 | c.7544C>T | p.Thr2515Ile | missense rs28897744 | ClinVar/LOVD | NA | 0.05 | 0.07 |
| CRC530 | Sigmoid colon cancer (34/F) | Ukrainian | Non | *APC* | - | - | c.5424_5426delCAA | p.Asn1808del | in-frame deletion rs587782002 | ClinVar | NA | 0,03 | NA |
| CRC597 | Descending colon cancer (43/M) | Russian | Non | *FANCE* | - | - | c.736_738delGGA | p.Gly246del | in-frame deletion rs45451605 | COSMIC/ClinVar | NA | 0,6 | 0,17 |
| CRC625 | Sigmoid colon cancer (48/M) | Belarusian | Father: laryngeal cancer | *TSC2* | - | - | c.4527_4529delCTT | p.Phe1510del | in-frame deletion rs137854239 | ClinVar | NA | 0,44 | 0,53 |

Abbreviations: NA – not available; M: male; F: female
