# Supplementary material for: Mutation Spectrum of Cancer-Associated Genes in Patients With Early Onset of Colorectal Cancer
Source: Front Oncol. 2019 Aug 2;9:673. doi: 10.3389/fonc.2019.00673 (PMC6688539; doi:10.3389/fonc.2019.00673)
Supplement: Supplementary file 4 [file Table_4.DOCX]

Table 4. The most significant mutations with deleterious effect found in the subgroup of patients with a family history of cancer

| Patient ID | Family history | Clinical features (age/gender/ethnicity) | Gene | Genotype | Mutation type | HGVSc/HGVSp | dbSNP ID | 1000G | Esp  6500 | ExAC | Database |
| --- | --- | --- | --- | --- | --- | --- | --- | --- | --- | --- | --- |
| CRC607 | Mat.great-grandmother: endometrial cancer;  mat. grandmother: gastric cancer;  Mat. aunt: CRC; Aunt of mother: gastric cancer;  Aunt of mother: CRC. | Ascending colon cancer (18/M/Kazakh) | *MSH2* | het | missense | c.2078G>A/p.Cys693Tyr | rs1057524909 | NA | NA | NA | ClinVar/LOVD |
|  |  |  | *MSH2* | het | missense | c.2072T>C/p.Ile691Thr | rs754824872 | NA | NA | NA | ClinVar |
|  |  |  | *TSC1* | het | missense | c.1460C>G/p.Ser487Cys | rs118203532 | 0.02 | 0.02 | 0.04 | ClinVar |
|  |  |  | *ERCC2* | het | missense | c.691G>A/p.Val231Met | rs200895828 | 0.06 | NA | 0.01 | ClinVar^#^ |
|  |  |  | *KIT* | het | missense | c.1352C>T/p.Ser451Phe | NA | NA | NA | NA | ClinVar/COSMIC |
| CRC613 | Family members with FAP and CRC | FAP; Primary multiple synchronous cancer. Cancer splenic flexure; Rectal cancer (39/F/Father: Russian; Mother: Kazakh) | *APC* | het | Stop-gain | c.4128T>G/p.Tyr1376Ter |  | NA | NA | NA | Novel^#^ |
|  |  |  | *MLH1* | het | missense | c.1852A>G/p.Lys618Glu | rs35001569 | 0.32 | 0.37 | 0.34 | ClinVar/LOVD^#^ |
|  |  |  | *FANCM* | het | missense | c.4881T>G/p.Phe1627Leu | NA | NA | NA | NA | LOVD |
| CRC2 | Family members with FAP and CRC | FAP; Sigmoid colon cancer (26/M/Turk) | *APC* | het | frameshift | c.3613delA/p.Ser1205AlafsTer60 | NA | NA | NA | NA | LOVD |
|  |  |  | *FANCI* | het | frameshift | c.3340delA/p.Thr1114ProfsTer50 | NA | NA | NA | NA | Novel |
|  |  |  | *MLH1* | *het* | missense | c.2146G>A/p.Val716Met | rs35831931 | 0.04 | 0.14 | 0.12 | ClinVar/LOVD |
| CRC629 | mat. grandmother and mother: OC | Primary multiple metachronous cancer. Rectosigmoid colon cancer; BC; OC (47/F/Russian) | *BRCA1* | het | frameshift | c.5329dupC/p.Gln1777ProfsTer74 | rs39750724; rs80357906 | NA | NA | 0.02 | LOVD, ClinVar |
| CRC621 | Mother: BC;  Father: cancer of the large duodenal papilla | Rectal cancer (47/F/Tatar) | *NBN* | het | missense | c.1670C>A/p.Ala557Asp | NA | NA | NA | NA | Novel |
|  |  |  | *FANCI* | het | missense | c.1813C>T/p.Leu605Phe | rs117125761 | 0.2 | 0.76 | 0.62 | ClinVar/LOVD |
|  |  |  | *NSD1* | het | missense | c.2456C>A/p.Thr819Asn | NA | NA | NA | NA | Novel |
| CRC587 | Father: kidney cancer | Rectal cancer (46/M/Kazakh) | *FANCD2* | het | missense | c.1306C>A/p.Leu436Met | rs373898927 | NA | 0.01 | NA | Novel^#^ |
|  |  |  | *PMS1* | het | missense | c.2780A>G/p.Tyr927Cys | rs111254723 | NA | NA | NA | Novel |
| CRC627 | pat. grand-father: lung cancer;  Pat. grandmother: BC | Rectal cancer (46/M/Russian) | *SLX4* | het | missense | c.1442G>A/p.Arg481Gln | rs145194745 | 0.02 | 0.01 | NA | Novel |
|  |  |  | *ATM* | het | missense | c.1229T>C/p.Val410Ala | rs56128736 | 0.08 | 0.18 | 0.22 | Novel^#^ |
| CRC625 | Father: laryngeal cancer | Sigmoid colon cancer (48/F/Belarusian) | *TSC2* | het | In-frame deletion | c.4527_4529delCTT/p.Phe1510del | rs137854239;rs13785439 | 0 | 0,44 | 0,53 | ClinVar |
|  |  |  | *MET* | het | missense | c.3029C>T/p.Thr1010Ile | rs56391007 | 0.34 | 0.89 | 0.79 | ClinVar^#^ |
| CRC584 | Father: gastric cancer | Rectal cancer (25/M/Kazakh) | *NSD1* | het | missense | c.3133C>T/p.Arg1045Cys | rs377148087 | NA | 0,01 | NA | ClinVar |
|  |  |  | *TSC2* | het | missense | c.3475C>T/p.Arg1159Trp | rs45517295 | 0.16 | NA | 0.04 | ClinVar |
| CRC626 | Mother: endometrial cancer | Rectalcancer(36/F/Kazakh) | *ATM* | het | missense | c.146C>G/p.Ser49Cys | rs1800054 | 0.42 | 0.99 | 0.74 | ClinVar/LOVD |
|  |  |  | *FANCA* | het | missense | c.1592A>G/p.Tyr531Cys | NA | NA | NA | NA | Novel |
| CRC632 | Father: gastric cancer | Rectal cancer (32/M/Kazakh) | *WRN* | het | missense | c.2983G>A/p.Ala995Thr | rs140768346 | 0.08 | 0.21 | 0.22 | ClinVar |
|  |  |  | *FANCI* | het | missense | c.1813C>T/p.Leu605Phe | rs117125761 | 0.2 | 0.76 | 0.62 | ClinVar/LOVD |
| CRC639 | Mother: rectal cancer | Sigmoid colon cancer (49/F/Russian) | *WRN* | het | missense | c.95A>G/p.Lys32Arg | rs34477820 | 0.12 | 0.3 | 0.37 | ClinVar/LOVD |
|  |  |  | *BRCA2* | het | missense | c.7544C>T/p.Thr2515Ile | rs28897744 | NA | 0.05 | 0.07 | ClinVar/LOVD |
| CRC368 | Mother: rectal cancer | Rectal cancer (39/F/Kazakh) | *FANCC* | het | missense | c.973G>A/p.Ala325Thr | rs201407189 | 0.14 | NA | 0.08 | ClinVar/LOVD^#^ |
|  |  |  | *FANCA* | het | missense | c.3031C>T/p.Arg1011Cys | rs142377616 | NA | 0.01 | 0.01 | ClinVar/LOVD |
| CRC335 | Father: prostate cancer | Rectosigmoid colon cancer (47/F/Russian) | *ATM* | het | missense | c.7429G>A/p.Gly2477Arg | rs778550056 | NA | NA | NA | Novel |
| CRC622 | mother: ascending colon cancer;  mat.grandmother: gastric cancer | Cecum cancer (43/M/Russian) | *MLH1* | het | missense | c.114C>G/p.Asn38Lys | rs267607706 | NA | NA | NA | LOVD, ClinVar |
| CRC640 | Mother: rectal cancer | Rectal cancer (40/F/Kazakh) | *TSC2* | het | missense | c.275A>T/p.Glu92Val | rs137853994 | 0.06 | 0.15 | 0.19 | ClinVar/LOVD |
| CRC596 | Family members with CRC and diffuse polyposis | PJS (20/M/German); Rectal cancer (33) | *APC* | het | Stop-gain | c.3827C>G/p.Ser1276Ter | rs1060503299 | NA | NA | NA | LOVD, ClinVar^#^ |

Abbreviations: NA – not available; OC - ovarian cancer; BC - breast cancer; mat. - maternal; pat. – paternal; Het – heterozygote; M - male; F – female; ^#^ - described in COSMIC
