# Supplementary material for: Mutation Spectrum of Cancer-Associated Genes in Patients With Early Onset of Colorectal Cancer
Source: Front Oncol. 2019 Aug 2;9:673. doi: 10.3389/fonc.2019.00673 (PMC6688539; doi:10.3389/fonc.2019.00673)
Supplement: Supplementary file 5 [file Table_5.DOCX]

Table 5. The most significant mutations with deleterious effect found in the subgroup of patients with primary multiple tumors

| Patient ID | Family history | Clinical features (age/gender/ethnicity) | Gene | Genotype | Mutation type | HGVSc/HGVSp | dbSNP ID | 1000G | Esp  6500 | ExAC | Database |
| --- | --- | --- | --- | --- | --- | --- | --- | --- | --- | --- | --- |
| CRC553 | No family history | Primary multiple metachronous cancer. Sigmoid colon cancer; Lung cancer (47/F/Russian) | *FANCI* | het | missense | c.3623T>A/p.Leu1208Gln | NA | NA | NA | NA | Novel |
|  |  |  | *BRCA2* | het | missense | c.10045A>G/p.Thr3349Ala | rs80358387 | NA | 0.02 | NA | ClinVar/LOVD |
|  |  |  | *CHEK2* | het | missense | c.308T>C/p.Phe103Ser | NA | NA | NA | NA | ClinVar |
|  |  |  | *FANCA* | het | missense | c.1874G>C/p.Cys625Ser | rs139235751 | 0.12 | 0.23 | 0.28 | ClinVar |
| CRC382 | No family history | Primary multiple synchronous cancer. Rectum cancer; Cancer of both adrenal glands (38/M) | *BRCA2* | het | frameshift variant | c.6304_6305delGT/p.Val2102IlefsTer8 | rs886040648 | NA | NA | NA | ClinVar |
|  |  |  | *FLCN* | het | missense | c.502C>T/p.Arg168Cys | rs587778367 | NA | NA | NA | ClinVar |
| CRC344 | No family history | Primary multiple synchronous cancer. Ascending colon cancer; Rectal cancer (46/F/German) | *BMPR1A* | het | frameshift variant | c.152delC/p.Ala51AspfsTer9 | NA | NA | NA | NA | Novel |
|  |  |  | *CHEK2* | het | missense | c.599T>C/p.Ile200Thr | rs17879961 | 0.1 | 0.16 | 0.41 | ClinVar^#^ |
| CRC624 | No family history | Primary multiple synchronous cancer. Sigmoid colon cancer; Hepatic flexure of the colon cancer (46/M/Russian) | *MLH1* | het | in-frame deletion | c.1852_1854delAAG/p.Lys618del | rs121912962 rs587782285 | NA | NA | NA | LOVD, ClinVar^#^ |
|  |  |  | *CEP57* | het | missense | c.333G>C/p.Gln111His | rs117321017 | 0.4 | 0.58 | 0.59 | ClinVar/LOVD |
| CRC139 | No family history | Primary multiple synchronous cancer. Rectosigmoid colon cancer; Cancer of the splenic flexure of the colon (46/F/Russian) | *FANCC* | het | missense | c.77C>T/p.Ser26Phe | rs1800361 | 0.26 | 0.5 | 0.47 | ClinVar/LOVD |
| CRC369 | No family history | Primary multiple metachronous cancer. Sigmoid colon cancer; Ascending colon cancer (47/M/Uigur) | *CYLD* | het | missense | c.806T>C/p.Met269Thr | NA | NA | NA | NA | Novel |
| CRC570 | No family history | Primary multiple metachronous cancer. Rectosigmoid colon cancer; Gastric cancer  (46/F/Kazakh) | *FANCC* | het | missense | c.584A>T/p.Asp195Val | rs1800365 | 0.26 | 0.31 | 0.31 | ClinVar/LOVD |
| CRC613 | Family members with FAP and CRC | FAP; Primary multiple synchronous cancer. Cancer splenic flexure; Rectal cancer (39/F/Father: Russian; Mother: Kazakh) | *APC* | het | Stop-gain | c.4128T>G/p.Tyr1376Ter |  | NA | NA | NA | Novel^#^ |
|  |  |  | *MLH1* | het | missense | c.1852A>G/p.Lys618Glu | rs35001569 | 0.32 | 0.37 | 0.34 | ClinVar/LOVD^#^ |
|  |  |  | *MLH1* | het | missense | c.1853A>C/p.Lys618Thr | rs63750449 | 0.32 | 0.38 | 0.34 | ClinVar/LOVD^#^ |
|  |  |  | *FANCM* | het | missense | c.4881T>G/p.Phe1627Leu | NA | NA | NA | NA | LOVD |
| CRC629 | mat. grandmother and mother: OC | Primary multiple metachronous cancer. Rectosigmoid colon cancer; BC; OC (47/F/Russian) | *BRCA1* | het | frameshift variant | c.5329dupC/p.Gln1777ProfsTer74 | rs39750724; rs80357906 | NA | NA | 0.02 | LOVD, ClinVar |

Abbreviations: NA – not available; Het – heterozygote; M - male; F – female, ^#^ - described in COSMIC
