# Supplementary material for: Mutation Spectrum of Cancer-Associated Genes in Patients With Early Onset of Colorectal Cancer
Source: Front Oncol. 2019 Aug 2;9:673. doi: 10.3389/fonc.2019.00673 (PMC6688539; doi:10.3389/fonc.2019.00673)
Supplement: Supplementary file 6 [file Table_6.DOCX]

Supplementary Table 6. The most significant mutations with deleterious effect found in the subgroup of patients with sporadic cases

| **Patient ID** | **Clinical features (age at diagnosis/gender)** | **Ethnicity** | **Gene** | **HGVSc** | **HGVSp** | **Consequence and dbSNP ID** | **Database** | **Population frequency** | | |
| --- | --- | --- | --- | --- | --- | --- | --- | --- | --- | --- |
|  |  |  |  |  |  |  |  | **1000G** | **Esp**  **6500** | **ExAC** |
| CRC382 | Rectum cancer (38/M) | Ukrainian | *BRCA2* | c.6304_6305delGT | p.Val2102IlefsTer8 | frameshift rs886040648 | ClinVar | NA | NA | NA |
| CRC442 | Rectosigmoid colon cancer (41/M) | Kazakh | *MSH6* | c.4068_4071dupGATT | p.Lys1358AspfsTer2 | frameshift rs55740729 | LOVD/ ClinVar | NA | NA | 0.23 |
|  |  |  | *RB1* | c.2777A>G | p.Glu926Gly | missense  NA | Novel | NA | NA | NA |
| CRC609 | Rectum cancer (40/M) | Kazakh | *APC* | c.907delA | p.Arg303GlyfsTer2 | frameshift  NA | Novel | NA | NA | NA |
|  |  |  | *NBN* | c.657_661delACAAA | p.Lys219AsnfsTer16 | frameshift rs587776650 | LOVD/ ClinVar | NA | 1.45 | 0.02 |
|  |  |  | *ATM* | c.6743dupA | p.Asp2249GlyfsTer24 | frameshift  NA | Novel | NA | NA | NA |
|  |  |  | *MSH2* | с.2542G>T | p.Ala848Ser | missense rs746972142 | ClinVar | NA | NA | NA |
| CRC380 | Cancer splenic flexure (23/M) | Uigur | *MUTYH* | c.721C>T | p.Arg241Trp | missense rs34126013 | LOVD/  ClinVar | NA | NA | 0.01 |
|  |  |  | *NSD1* | c.1135G>A | p.Ala379Thr | missense NA | Novel | NA | NA | NA |
|  |  |  | *FANCM* | c.4931G>A | p.Arg1644Gln | missense rs138151018 | ClinVar | 0.32 | 0.01 | 0.15 |
|  |  |  | *PMS2* | c.751G>A | p.Val251Met | rs142434011 |  | NA | 0.01 | NA |
| CRC545 | Rectum cancer (43/M) | Kazakh | *BRCA2* | c.9976A>T | p.Lys3326Ter | stop-gain rs11571833 | LOVD/ ClinVar^#^ | 0.44 | 0.65 | 0.7 |
| CRC616 | Sigmoid colon cancer (17/F) | Kazakh | *APC* | c.4128T>G | p.Tyr1376Ter | stop-gain  NA | Novel^#^ | NA | NA | NA |
| CRC628 | Rectum cancer (43/M) | Kazakh | *DICER1* | c.4991C>A | p.Ser1664Ter | stop-gain  NA | Novel | NA | NA | NA |
|  |  |  | *BRCA2* | c.6100C>T | p.Arg2034Cys | missense rs1799954 | ClinVar/  LOVD/  COSMIC | 0.14 | 0.4 | 0.32 |
| CRC581 | Rectum cancer (49/F) | Kazakh | *BRCA1* | c.5341-2delA | Unknown | splice acceptor rs878853285 | ClinVar | NA | NA | NA |
|  |  |  | *NF2* | c.1439C>T | p.Thr480Met | missense rs145666157 | ClinVar/  COSMIC | 0.04 | NA | 0.04 |
| CRC558 | Сecum cancer (37/M) | Russian | *FANCI* | c.2889+1G>A | Unknown | splice donor rs1556861311 | ClinVar | NA | NA | NA |
|  |  |  | *DICER1* | c.3295A>G | p.Lys1099Glu | missense  NA | ClinVar | NA | NA | NA |
| CRC589 | Rectosigmoid colon cancer (46/M) | Tatar | *BRCA2* | c.6937+1G>A | Unknown | splice donor rs886040935 | ClinVar# | NA | NA | NA |
|  |  |  | *FANCC* | c.77C>T | p.Ser26Phe | missense rs1800361 | ClinVar/  LOVD | 0.26 | 0.5 | 0.47 |
| CRC6 | Rectosigmoid colon cancer (39/F) | Kazakh | *BRCA2* | c.8187G>T | p.Lys2729Asn | missense rs80359065 | ClinVar | 0.26 | NA | 0.08 |
|  |  |  | *ATM. C11ORF65* | c.5975A>C | p.Lys1992Thr | missense rs150757822 | ClinVar/  LOVD | 0.02 | 0.02 | 0.04 |
| CRC82 | Rectum cancer (48/F) | Russian | *EPCAM* | c.831A>G | p.Ile277Met | missense rs115283528 | ClinVar/  LOVD | 0.08 | 0.18 | 0.23 |
| CRC95 | Rectum cancer (44/M) | Russian | *BRCA2* | c.5590G>A | p.Asp1864Asn | missense rs587781536 | ClinVar | NA | NA | NA |
| CRC174 | Transverse colon cancer (36/F) | Kazakh | *PTCH1* | c.2956G>C | p.Asp986His | missense  NA | Novel | NA | NA | NA |
|  |  |  | *TSC1* | c.819T>G | p.Asp273Glu | missense rs148756522 | ClinVar | 0.02 | 0.01 | NA |
| CRC205 | Rectum cancer (49/M) | Uigur | *NBN* | c.1690G>A | p.Glu564Lys | missense rs72550742 | ClinVar | 0.24 | NA | 0.1 |
|  |  |  | *ATM* | c.2932T>C | p.Ser978Pro | missense rs139552233 | ClinVar/  LOVD/  COSMIC | 0.06 | 0.03 | 0.12 |
|  |  |  | *MET* | c.3029C>T | p.Thr1010Ile | missense rs56391007 | ClinVar/  COSMIC | 0.34 | 0.89 | 0.79 |
|  |  |  | *XPC* | c.203A>T | p.Asp68Val | missense rs56012223 |  | 0.04 | NA | 0.03 |
| CRC283 | Rectum cancer (29/F) | Kazakh | *FANCM* | c.2996C>T | p.Pro999Leu | missense rs148304968 | ClinVar/  COSMIC | 0.08 | 0.07 | 0.03 |
|  |  |  | *PALB2* | c.2360C>T | p.Thr787Ile | missense rs201042302 | ClinVar/  LOVD | 0.02 | NA | NA |
| CRC330 | Sigmoid colon cancer (24/M) | Kazakh | *MSH6* | c.2408A>G | p.Asp803Gly | missense rs63751450 | ClinVar/  LOVD | 0.02 | 0.02 | 0.01 |
| CRC385 | Sigmoid colon cancer (50/F) | German | *BRCA1* | c.4039A>G | p.Arg1347Gly | missense rs28897689 | ClinVar/  LOVD | 0.06 | 0.48 | 0.4 |
| CRC44 | Rectum cancer (49/F) | Kazakh | *PALB2* | c.1748T>G | p.Leu583Trp | missense rs587782151 | ClinVar | NA | NA | NA |
| CRC137 | Sigmoid colon cancer (34/M) | Russian | *VHL* | c.74C>T | p.Pro25Leu | missense rs35460768 | ClinVar/  LOVD/  COSMIC | 0.04 | 0.29 | 0.52 |
|  |  |  | *AIP* | c.911G>A | p.Arg304Gln | missense rs104894190 | ClinVar. LOVD | NA | 0.07 | 0.14 |
| CRC238 | Rectosigmoid colon cancer (29/M) | Kazakh | *RET* | c.3112A>G | p.Thr1038Ala | missense rs201740483 | ClinVar/  LOVD/  COSMIC | 0.22 | 0.28 | 0.41 |
|  |  |  | *XPC* | c.1443G>T | p.Lys481Asn | missense rs182616621 | ClinVar | 0.22 | 0.28 | 0.41 |
| CRC335 | Rectosigmoid colon cancer (47/F) | Russian | *ATM* | c.7429G>A | p.Gly2477Arg | missense rs778550056 | Novel | NA | NA | NA |
| CRC438 | Rectosigmoid colon cancer (49/F) | Kazakh | *XPC* | c.872C>G | p.Ser291Cys | missense rs184879571 | ClinVar | 0.16 | 0.07 | 0.28 |
|  |  |  | *DICER1* | c.2540C>G | p.Thr847Arg | missense  NA | Novel | NA | NA | NA |
| CRC530 | Sigmoid colon cancer (34/F) | Ukrainian | *WRN* | c.95A>G | p.Lys32Arg | missense rs34477820 | ClinVar/  LOVD | 0.12 | 0.3 | 0.37 |
| CRC544 | Sigmoid colon cancer (49/F) | Russian | *WRN* | c.2059T>G | p.Leu687Val | missense rs185468906 | ClinVar | 0.08 | NA | 0.1 |
|  |  |  | *RET* | c.129C>A | p.Asp43Glu | missense  NA | Novel | NA | NA | NA |
| CRC546 | Sigmoid colon cancer (38/F) | Father:Uzbek; Mother: Kazakh | *ATM* | c.4388T>G | p.Phe1463Cys | missense rs138327406 | ClinVar | 0.04 | 0.11 | 0.14 |
|  |  |  |  | c.1810C>T | p.Pro604Ser | missense  rs2227922 | ClinVar | 0.26 | 0.4 | 0.31 |
| CRC551 | Sigmoid colon cancer (50/M) | Kazakh | *MET* | c.632T>G | p.Leu211Trp | missense rs45483396 | ClinVar | 0.04 | NA | 0.03 |
|  |  |  | *BRIP1* | c.1902G>C | p.Gln634His | missense rs1060501748 | ClinVar | NA | NA | NA |
| CRC569 | Sigmoid colon cancer (36/F) | Kazakh | *FANCA* | c.184C>T | p.Leu62Phe | missense  NA | Novel | NA | NA | NA |
| CRC579 | Rectum cancer (45/M) | Uigur | *PALB2* | c.3296C>G | p.Thr1099Arg | missense rs142132127 | ClinVar | 0.02 | NA | 0.01 |
| CRC586 | Sigmoid colon cancer (37/M) | Kazakh | *CDH1* | c.2494G>A | p.Val832Met | missense rs35572355 | ClinVar | 0.04 | 0.02 | 0.02 |
| CRC592 | Sigmoid colon cancer (40/M) | Korean | *BLM* | c.2293G>A | p.Val765Ile | missense rs191789336 | ClinVar | 0.02 | NA | 0.03 |
| CRC593 | Sigmoid colon cancer (36/F) | Russian | *PRKAR1A* | c.287G>T | p.Arg96Leu | missense  NA | Novel | NA | NA | NA |
| CRC594 | Rectum cancer (42/F) | Kazakh | *MLH1* | c.649C>T | p.Arg217Cys | missense rs4986984 | ClinVar | 0.06 | NA | 0.03 |
| CRC599 | Sigmoid colon cancer (32/F) | Kazakh | *BLM* | c.2693G>A | p.Arg898Lys | missense  NA | Novel | NA | NA | NA |
|  |  |  | *ATM* | c.5558A>T | p.Asp1853Val | missense rs1801673 | ClinVar/  LOVD/  COSMIC | 0.18 | 0.48 | 0.52 |
|  |  |  | *DICER1* | c.484G>T | p.Gly162Cys | missense rs1801673 | Novel | NA | NA | NA |
| CRC600 | Rectum cancer (45/M) | Kazakh | *MSH2* | c.1031A>C | p.Gln344Pro | missense  NA | Novel | NA | NA | NA |
|  |  |  | *DICER1* | c.1493T>G | p.Phe498Cys | missense  NA | Novel | NA | NA | NA |
| CRC601 | Rectum cancer (38/M) | Dungan | *RET* | c.874G>A | p.Val292Met | missense rs34682185 | ClinVar | 0.38 | NA | 0.05 |
|  |  |  | *FANCM* | c.4931G>A | p.Arg1644Gln | missense rs138151018 | ClinVar | 0.32 | 0.01 | 0.15 |
|  |  |  | *MSH2* | c.1168C>T | p.Leu390Phe | missense rs17224367 | ClinVar/  LOVD | 0.28 | 0.01 | 0.16 |
| CRC602 | Cecum cancer (40/F) | Kazakh | *BRCA2* | c.5070A>C | p.Lys1690Asn | missense rs56087561 | ClinVar/  LOVD | NA | 0.02 | 0.02 |
|  |  |  | *NSD1* | c.1865G>C | p.Cys622Ser | missense  NA | Novel | NA | NA | NA |
| CRC605 | Sigmoid colon cancer (45/M) | Uigur | *MSH2* | c.1882G>C | p.Gly628Arg | missense rs371776176 | ClinVar | NA | NA | NA |
|  |  |  | *CDH1* | c.2494G>A | p.Val832Met | missense rs35572355 | ClinVar | 0.04 | 0.02 | 0.02 |
|  |  |  | *ERCC4* | c.2734G>A | p.Gly912Arg | missense rs150077735 | ClinVar | 0.02 | NA | 0.02 |
| CRC606 | Rectum cancer (47/M) | Kazakh | *MSH6* | c.3488A>T | p.Glu1163Val | missense rs63750252 | ClinVar/  LOVD/COSMIC | 0.28 | NA | 0.12 |
|  |  |  | *MSH2* | c.1168C>T | p.Leu390Phe | missense rs17224367 | ClinVar/  LOVD | 0.28 | 0.01 | 0.16 |
|  |  |  | *NBN* | c.511A>G | p.Ile171Val | missense rs61754966 | ClinVar/  LOVD | 0.04 | 0.12 | 0.14 |
| CRC611 | Rectum cancer (43/M) | Kazakh | *BRCA2* | c.7544C>T | p.Thr2515Ile | missense rs28897744 | ClinVar/  LOVD | NA | 0.05 | 0.07 |
| CRC612 | Rectosigmoid colon cancer (38/F) | Russian | *FANCC* | c.77C>T | p.Ser26Phe | missense rs1800361 | ClinVar | 0.26 | 0.5 | 0.47 |
| CRC618 | Rectum cancer (47/F) | Russian | *RB1* | c.2392C>T | p.Arg798Trp | missense rs187912365 | ClinVar | 0.08 | 0.01 | 0.01 |
| CRC620 | Rectum cancer (47/F) | Kazakh | *BRCA2* | c.3349A>G | p.Ile1117Val | missense rs397507307 | ClinVar/  LOVD | NA | NA | NA |
|  |  |  | *DICER1* | c.1381A>G | p.Ile461Val | missense rs141163928 | ClinVar | NA | 0.02 | 0.01 |
| CRC623 | Rectum cancer (32/M) | Azerbajanian | *WRN* | c.2983G>A | p.Ala995Thr | missense rs140768346 | ClinVar | 0.08 | 0.21 | 0.22 |
| CRC630 | Sigmoid colon cancer (30/F) | Kazakh | *AIP* | c.47G>A | p.Arg16His | missense rs145047094 | ClinVar/  LOVD | 0.06 | 0.25 | 0.2 |
| CRC635 | Hepatic flexure of the colon cancer (37/M) | Father: Bashkir; Mother: Kazakh | *PMS1* | c.278G>A | p.Arg93His | missense rs778185859 | Novel | NA | NA | NA |
|  |  |  | *RET* | c.937C>T | p.Arg313Trp | missense  NA | ClinVar | NA | NA | NA |
| CRC638 | Rectum cancer (44/M) | Kazakh | *XPC* | c.281G>T | p.Ser94Ile | missense  NA | Novel | NA | NA | NA |
|  |  |  | *ATM* | c.107A>G | p.Asp36Gly | missense  NA | ClinVar/  LOVD | NA | NA | NA |
|  |  |  | *NSD1* | c.7852G>A | p.Val2618Ile | missense  rs373787813 |  | 0.02 | 0.02 | 0.01 |
| CRC420 | Rectum cancer (37/M) | Kazakh | *XPC* | c.1443G>T | p.Lys481Asn | missense  rs182616621 | ClinVar | 0.22 | 0.28 | 0.41 |
| CRC457 | Rectum cancer (47/M) | Kazakh | *BMPR1A* | c.316T>C | p.Ser106Pro | missense  NA | Novel | NA | NA | NA |
| CRC529 | Rectum cancer (47/F) | Kazakh | *FANCA* | c.3031C>T | p.Arg1011Cys | missense rs142377616 | ClinVar/  LOVD | NA | 0.01 | 0.01 |
| CRC548 | Rectum cancer (40/F) | Kazakh | *XPC* | c.923C>T | p.Ala308Val | missense  NA | Novel | NA | NA | NA |
| CRC573 | Sigmoid colon cancer (32/F) | Russian | *MET* | c.3029C>T | p.Thr1010Ile | missense rs56391007 | ClinVar/  COSMIC | 0.34 | 0.89 | 0.79 |
|  |  |  | *RET* | c.1717G>A | p.Val573Met | missense rs758766818 | Novel/  COSMIC | NA | NA | NA |
| CRC585 | Rectum cancer (30/F) | Kazakh | *FANCD2* | c.78A>C | p.Gln26His | missense rs45510294 | ClinVar/  LOVD | 0.02 | 0.06 | 0.06 |
| CRC603 | Rectum cancer (48/M) | Kazakh | *MSH6* | c.1481C>T | p.Ala494Val | missense  NA | ClinVar | NA | NA | NA |
|  |  |  | *ATM* | c.5558A>T | p.Asp1853Val | missense rs1801673 | ClinVar/  LOVD/  COSMIC | 0.18 | 0.48 | 0.52 |
| CRC610 | Rectum cancer (43/M) | Kazakh | *BRIP1* | c.430G>A | p.Ala144Thr | missense rs116952709 | ClinVar/  COSMIC | 0.46 | 0.02 | 0.13 |
| CRC641 | Sigmoid colon cancer (45/M) | Russian | *NF2* | c.1439C>T | p.Thr480Met | missense rs145666157 | ClinVar/  COSMIC | 0.04 | 0 | 0.04 |
| CRC101 | Rectum cancer (42/F) | Uigur | *BRCA2* | c.5590G>A | p.Asp1864Asn | missense rs587781536 | ClinVar | NA | NA | NA |
| CRC526 | Rectum cancer (41/M) | Russian | *NBN* | c.511A>G | p.Ile171Val | missense rs61754966 | ClinVar/  LOVD | 0.04 | 0.12 | 0.14 |
| CRC520 | Sigmoid colon cancer (34/M) | Russian | *FANCL* | c.108C>G | p.Phe36Leu | missense rs149726602 | ClinVar/  COSMIC | 0.18 | 0.02 | 0.13 |
| CRC547 | Sigmoid colon cancer (47/F) | Russian | *PRF1* | c.755A>G | p.Asn252Ser | missense rs28933375 | ClinVar/  LOVD/  COSMIC | 0.76 | 0.85 | 0.52 |
| CRC580 | Sigmoid colon cancer (41/F) | Kazakh | *NBN* | c.511A>G | p.Ile171Val | missense rs61754966 | ClinVar/  LOVD | 0.04 | 0.12 | 0.14 |

Abbreviations: NA – not available; M: male; F: female; F – female; ^#^ - described in COSMIC
